# Small molecule drug discovery for Ebola virus disease

**DOI:** 10.1039/d5md00533g

**Published:** 2025-08-06

**Authors:** Destiny Durante, Venkatesh Murugesh, Tyler Kalanquin, Irina N. Gaisina, Lijun Rong, Terry W. Moore

**Affiliations:** a Department of Pharmaceutical Sciences, Retzky College of Pharmacy, University of Illinois Chicago IL 60612 USA twmoore@uic.edu; b UICENTRE for Drug Discovery, University of Illinois Chicago IL 60612 USA; c Department of Microbiology and Immunology, College of Medicine, University of Illinois Chicago IL 60612 USA; d University of Illinois Cancer Center, University of Illinois Chicago IL 60612 USA

## Abstract

Known for its widespread outbreaks, including the 2013–2016 epidemic that infected almost 29 000 individuals and resulted in approximately 11 300 deaths, Ebola virus (EBOV) and related filoviruses remain a current threat as consecutive filoviral outbreaks have occurred between 2021 through 2025. Due to high fatality rates of 40–90% among infected individuals, researchers have invested significant efforts to discover effective treatments for Ebola virus disease. Small molecules hold great potential for treating Ebola virus disease because they can target various stages of the filoviral life cycle, such as entry, transcription, replication, and egress; however, the FDA has not yet approved any small molecule treatments for EBOV. In this review, we report both historic and recent progress in the discovery of small molecule drugs for EBOV.

## Abstract 2

Known for its widespread outbreaks, including the 2013–2016 epidemic that resulted in around 11 300 deaths, Ebola virus (EBOV) and related filoviruses remain a current threat as consecutive filoviral outbreaks have occurred between 2021 through 2025.

## Graphical abstract

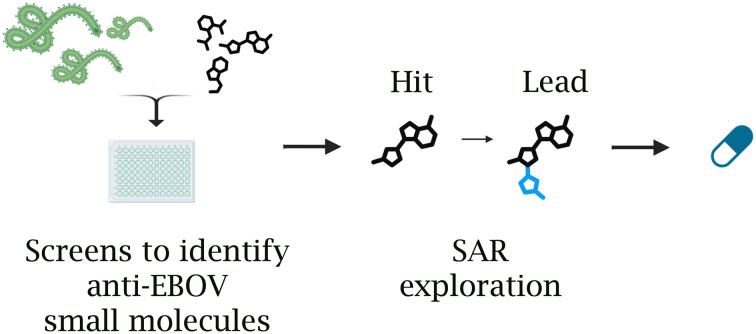

## EBOV background

### EBOV taxonomy, disease, and recent filoviral outbreaks

Since its 1976 discovery, Ebola virus (EBOV) has commanded worldwide attention as a pathogen of significant concern. As a member of the *Filoviridae* family and *Orthoebolavirus* genus, EBOV is a zoonotic RNA virus.^1^ The likely reservoir host is the fruit bat, which infects other animals including non-human primates.^2^ EBOV transmits from these animals to humans, and contact with infected bodily fluids, like saliva, sweat, blood, and semen, causes human-to-human infection.^3^ Other *Orthoebolavirus* species that are noted to be human-infectious include Sudan virus (SUDV), Bundibugyo virus (BDBV), and Taï Forest virus (TAFV). Reston virus (RESTV) infections have been reported without evidence of disease progression, while Bombali virus (BOMV) lacks documented cases of human infection.^4,5^

After successful viral transmission, EBOV infection rapidly leads to Ebola virus disease (EVD). EBOV targets a diverse range of host cells,^6–8^ primarily dendritic, monocyte, and macrophage cells. Immune cell susceptibility disrupts host immune responses by MAPK inhibition and cytokine production.^9,10^ Endothelial cells are targeted during the late stages of EBOV infection, which causes vascular leakage and hemorrhaging due to reduced levels of blood coagulation factors.^6,11,12^ As a result, EVD rapidly progresses from mild viral symptoms like fever, fatigue, muscle pain, and sore throat in days 4–7, to more advanced symptoms like internal and external bleeding and organ failure in the latter stages of infection.^13–16^ The quick onset of severe symptoms associated with EVD often results in high fatality rates, averaging around 40%.^17,18^

Isolated cases of infection have historically triggered extensive outbreaks, with most taking place in Central and Western African countries.^17,19^ The 2013–2016 EBOV epidemic was the largest and most fatal outbreak, with nearly 29 000 reported cases and approximately 11 300 deaths. The main countries impacted by the epidemic included Guinea, Sierra Leone, and Liberia; however, cases were also reported in additional countries like Nigeria, Spain, the United Kingdom, and the United States. The second largest EBOV outbreak occurred shortly after the epidemic, mainly impacting the Democratic Republic of Congo during 2018–2020. Subsequently, there have been several consecutive outbreaks, including those from EBOV in Uganda during 2021; SUDV in Guinea during 2022; Marburg virus (MARV), a related filovirus, in Guinea during 2023; MARV in Rwanda during 2024; and the most recent 2025 SUDV outbreak in Uganda that ended in April. Increased EBOV and related viral outbreaks in recent consecutive years stresses the need for effective filoviral therapeutics.

### EBOV biology

**Fig. 1:**
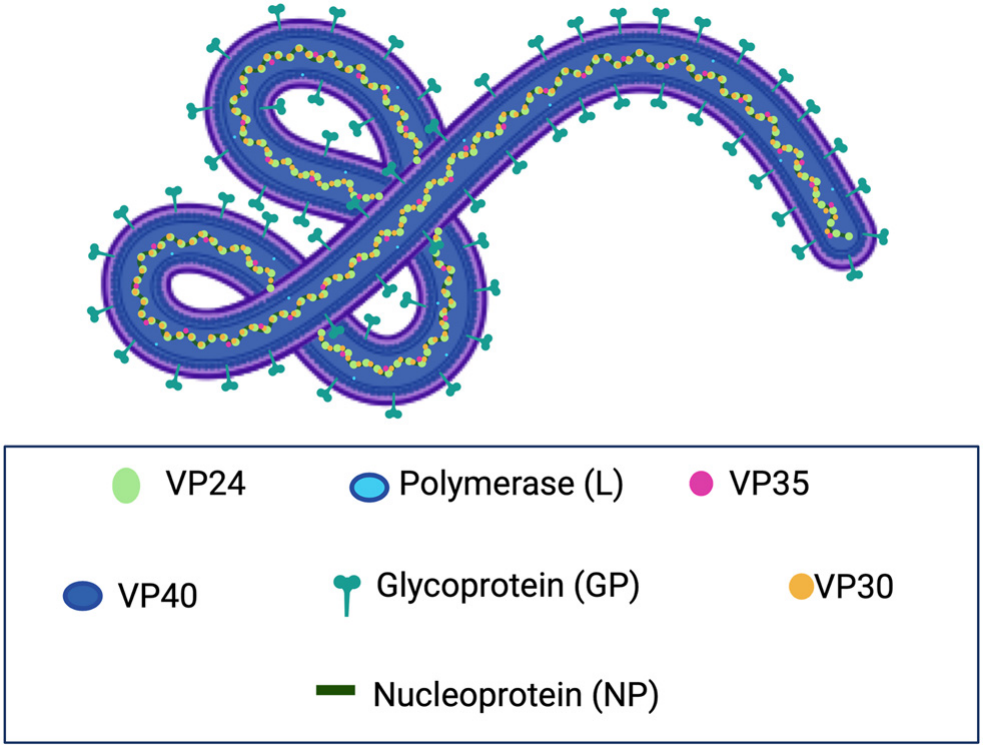
Illustration of Ebola virus (EBOV) and viral proteins GP, L, NP, VP24, VP40, VP35, and VP30.

**Fig. 2:**
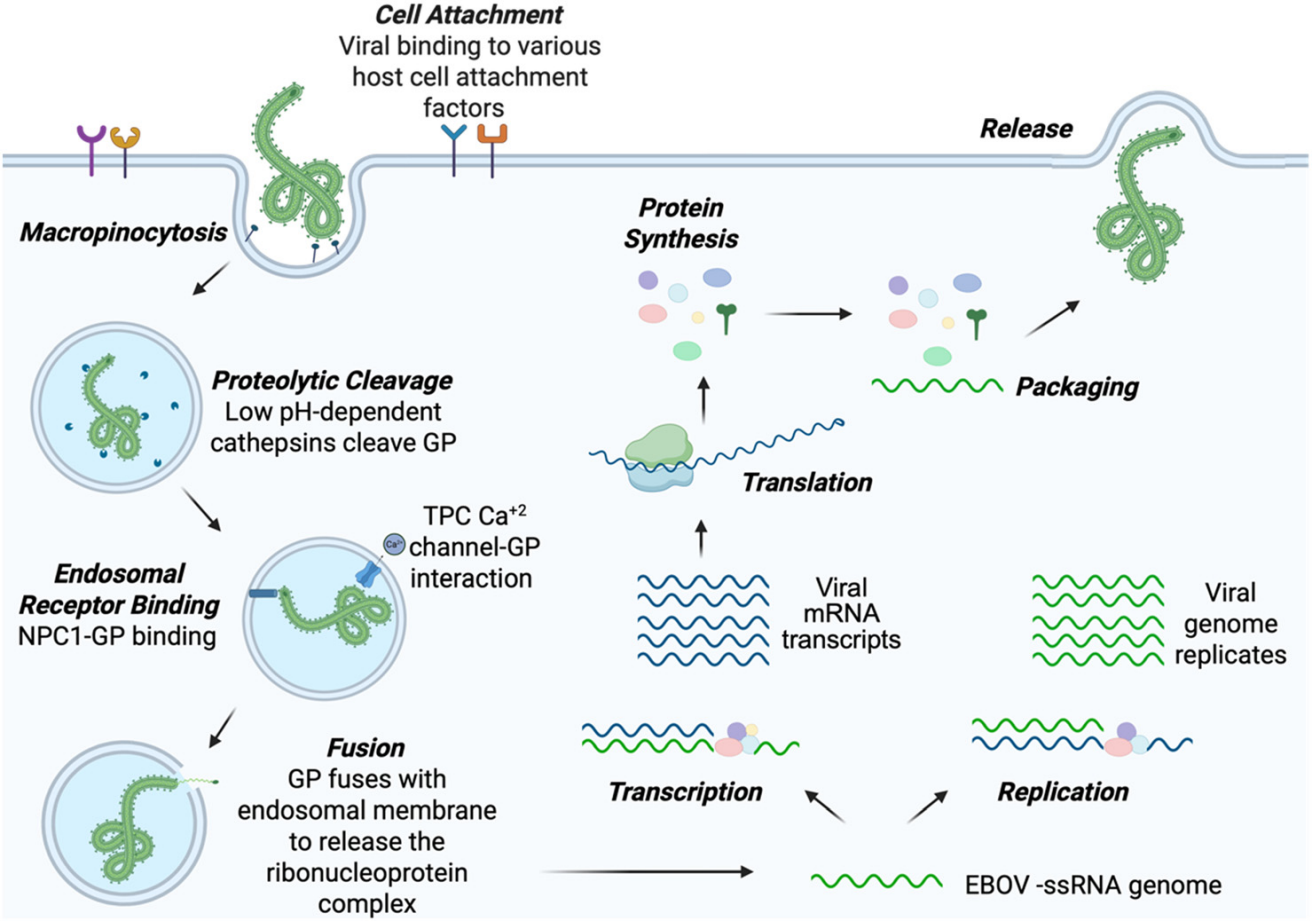
Schematic overview of Ebola virus (EBOV) life cycle that includes cell attachment, macropinocytosis, proteolytic cleavage, endosomal receptor binding, fusion, transcription, replication, translation, packaging, and release. GP: glycoprotein; NPC1: Niemann–Pick disease type C1; TPC: two-pore channel.

EBOV is an enveloped virus containing a negative-sense, single-stranded RNA genome that is approximately 19 kilobases long and encodes seven genes: the glycoprotein (GP), nucleoprotein (NP), VP24, VP30, VP35, VP40, and RNA polymerase (L) (Fig. 1).^20^ GP is expressed on the viral surface and is an essential protein for mediating viral entry into the host cell. As a homotrimer, each GP monomer is composed of a GP_1_ and GP_2_ subunit. GP_1_ determines host tropism and facilitates viral-cellular attachment by promiscuously binding to various host-cell receptors including β1 integrins, C-type lectins, T-cell immunoglobulin and mucin domain 1, glycosaminoglycans, and tyrosine kinase receptors (Fig. 2).^21–25^ EBOV uses the heavily glycosylated GP_1_ subunit to bind to receptors that recognize N- and O-linked oligosaccharides. Once bound to the cell, EBOV is macropinocytosed at the surface and trafficked to the endosome.^26^ As the endosome progresses toward maturity, the vesicles increase in acidity, which activates low pH-induced cysteine proteases Cathepsin L and B that proteolytically cleave the mucin-like domain and glycan cap from GP_1_.^27^ This cleavage reveals the receptor-binding site (RBS) within GP and enables endosomal receptor Niemann–Pick C1 (NPC1) to bind at this region.^28^ This major binding event, in addition to GP interactions with Ca^2+^ and two-pore channels (TPCs), triggers fusion, where GP undergoes conformational changes that are mediated by GP_2_.^29,30^ During this transformation, the newly folded GP creates a pore within the host endosomal membrane, which allows for the release of the viral ribonucleoprotein complex into the host cytoplasm for viral genome replication, protein synthesis, and the production of viral progeny.

Proteins VP30, VP35, and L form a complex with NP to complete viral transcription and replication. VP30 is an innately phosphorylated transcriptional activator. To initiate transcription, VP30 must be dephosphorylated by host phosphatases;^31,32^ however, replication can still occur while VP30 is in the phosphorylated state. L is activated by host factors like DNA topoisomerase and heat shock proteins for polymerase activity.^33,34^ VP35 protects viral dsRNA by capping the ends, preventing recognition and degradation by host cell RIG-I-helicase.^35–37^ Sufficient numbers of RNA transcripts and replicates lead to the formation of inclusion bodies within the host cytoplasm that are enriched with the viral genome and proteins.^38^

Both VP35 and VP24 provide viral protection from host factors by antagonizing host interferon (IFN) responses.^38^ VP35 inhibits host dsRNA sensing, IFN gene expression, and IFN activity.^39^ MAPK and NF-κB pathway activation are inhibited by VP24.^40^ In addition, VP24 also assists VP40 in the packaging of the newly synthesized viral genome and proteins. VP40 further regulates the intracellular transport of packaged viral components to the inner leaflet of the plasma membrane for viral egress and the release of new progeny.^41,42^

### *In vitro* systems to study EBOV and identify inhibitors

High pathogenicity and lethality of EBOV require viral containment in biosafety level 4 (BSL 4) facilities. Heavy restrictions on infectious EBOV can limit the study of filoviruses; however, surrogate systems or pseudotyped viruses are often used to study filoviral entry mechanisms. The pseudotyped viral system uses a glycoprotein of interest and an engineered viral vector that acts as the surrogate. In the case of EBOV study, human immunodeficiency virus (HIV) and vesicular stomatitis virus (VSV) are common surrogates that can incorporate EBOV GP on the viral surface, which allows the pseudovirus to maintain filoviral GP-dependent entry mechanisms.^43^ During plasmid generation, select coding regions of the surrogate viral genome are deleted so that produced viruses are defective in viral replication,^43^ while addition of reporter genes allows for the visualization and quantification of pseudoviral entry. For pseudoviral production, producer cells are transfected with plasmids containing the recombinant surrogate viral genome and filoviral GP.^44^

Pseudotyped systems are great models for filoviral entry; however, the inability of the pseudovirus to replicate confines their study to the entry process only, as seen with the HIV pseudovirus (HIV-EBOV GP). Unlike HIV, VSV is not pathogenic to humans; therefore, plasmids containing the full-length VSV genome are used, replacing VSV G with EBOV GP.^45^ The recombinant vectors are transfected in producer cells to generate replication-competent VSV pseudovirus (rVSV-EBOV GP) that has proven useful in evaluating drug resistance *via* escape mutants for EBOV.^46–48^ The challenge of viral similarity still remains as HIV and VSV are morphologically different from filamentous EBOV, and these differences have been shown to impact viral infectivity.^49^ VSV can also enter at the cell surface and bypass trafficking to the endosome, which is an essential step in filoviral entry.

Alternatively to the pseudovirus system, researchers found that expression of VP40 alone in transfected cells leads to the formation of filamentous EBOV virus-like particles (eVLP).^50,51^ EBOV VLP formation is solely based on VP40's ability to mediate viral budding in the host cell. Expression of both EBOV VP40 and GP generates eVLPs that model filoviral entry. Further incorporation of a mini-genome has proven useful, as mini-genomes are complementary DNA (cDNA) constructs of full-length or truncated viral RNA genomes. The cDNA is designed to encode a promoter, reporter gene, and viral 3′ and 5′ untranslated regions needed for viral transcription, replication, and packaging.^52,53^ Use of the mini-genome system that includes EBOV VP40 and GP genes generates transcription- and replication-competent virus-like particles (tr-VLP). Use of tr-VLPs thereby enables the study of all aspects of the EBOV life cycle and provides a screening method to assess therapeutic agents that target filoviral entry, replication, transcription, and egress.^52,54^

### Current EBOV therapeutic agents and value of small molecule treatments

Efforts for EBOV drug development have included siRNA therapeutics, ion channel inhibitors, combination therapies, peptides, antibodies, and small molecules.^55–64^ In 2019, the FDA approved the prophylactic agent, Ervebo, as it was shown to protect individuals, who were previously exposed to EBOV, from EVD.^65^ Although effective, Ervebo lacks cross-protective efficacy and is only approved for use against one orthoebolavirus species, Zaire. Furthermore, the EBOV vaccine is only indicated for one-time use, which exemplifies the need for available therapeutics in the event of future EBOV exposure.

In addition to the vaccine, there are two FDA-approved monoclonal antibody treatments for EBOV, Inmazeb and Ebanga.^66^ Both monoclonal antibody treatments target early steps of EBOV entry by neutralizing GP and recruiting immune cells to sites of infection. Due to their ease of synthesis, transport, and storage, small molecules are advantageous compared to antibody therapeutics because they can target several steps throughout the filoviral life cycle including entry, transcription, replication, and budding. In this review, we discuss recent advances of small-molecule therapeutic agents against EBOV to summarize and inspire innovation of novel antifiloviral treatments.

## Entry inhibitors

### Cell attachment

The first step in EBOV entry is host-cell attachment. Instead of targeting a specific receptor, EBOV promiscuously binds to various host attachment factors including β1 integrins, C-type lectins, T-cell immunoglobulin and mucin domain 1, and Tyrosine kinase receptors.^21–24^ EBOV uses the heavily glycosylated GP_1_ subunit to bind to receptors that recognize N- and O-linked oligosaccharides.

Heparan sulfate and heparin are cell-surface glycosaminoglycans composed of repeating glucosamine and uronic acid disaccharide units. They are responsible for regulating a range of biological activity, including filoviral/host attachment.^67^ Extosin 1, a host glycotransferase involved in the biosynthesis of heparan sulfate, was identified as a host factor involved in filoviral entry through the screening of genome-wide RNAi's.^25^ ELISA was used to demonstrate binding of heparan sulfate and heparin to EBOV GP. Pseudotyped and infectious EBOV entry were inhibited in A549 and human pulmonary artery endothelial cells upon treatment with various glycosaminoglycans. More recently, heparan sulfate was also shown to mediate EBOV infection in Caco-2 cells.^68^ These results indicate the usefulness of inhibiting viral-host attachment as a therapeutic mechanism.

In the case of C-type lectins, DC-SIGN^69^ is a known EBOV attachment factor that is specific to dendritic cells. DC-SIGN is a transmembrane receptor with four subunits that each contain a carbohydrate recognition domain (CRD). Thorough DC-SIGN investigation led to the development of multivalent glycoconjugate systems that bind to each CRD with high affinity. In the case of calix[4]arene glycoconjugates, researchers linked α-l-fucose or α-d-mannose with hydroxamic acid or pseudopeptide groups to a calixarene scaffold (Table 1).^70^ Binding of **glycoconjugates**1 and 2 to DC-SIGN's extracellular domain was confirmed *via* SPR, which assisted the ability to inhibit pseudotyped EBOV infection in Jurkat cells. α-l-fucose was the preferred binder, demonstrated by the EC_50_ of 289 nM for **glycoconjugate**1, compared to 634 nM for **glycoconjugate**2. Although these glycoconjugates are water-soluble, previous studies identified cytotoxicity as a major shortcoming of multivalent systems, as they have been shown to accumulate in cellular compartments and cause adverse effects.^71^ To address this, poly-l-lysine multivalent glycoconjugates that coupled d-mannose residues to lysine linkers were developed.^72^ In a flow cytometry experiment, **glycoconjugate**1d inhibited EBOV GP binding to B-THP cells expressing DC-SIGN at 0.198 nM. By labeling the active poly-l-lysine glycoconjugates with the pH-sensitive fluorescent dye rhodamine, researchers could visualize the presence of these active inhibitors in acidic compartments, which suggests that glycoconjugate binding reduces the presence of DC-SIGN on the host surface, further inhibiting attachment of EBOV. The fluorescent glycoconjugates were also cleared from the cells in a time-dependent manner within 24 hours, improving the potential cytotoxic effects.

**Table 1:** Names, structures, and references of EBOV entry inhibitors that target cellular attachment

Name	Structure	Ref
**Glycoconjugate**1	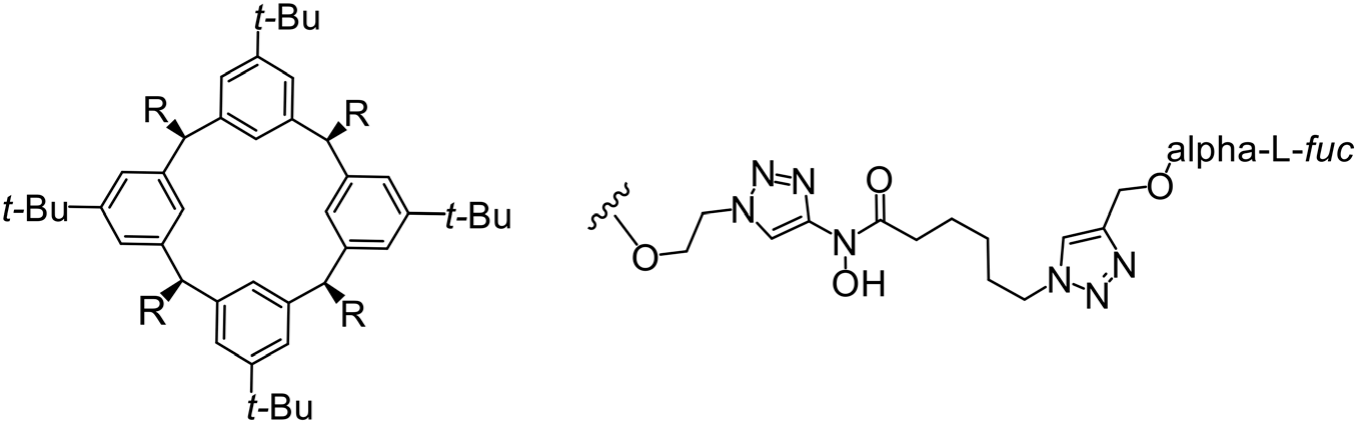	70
**Glycoconjugate**2	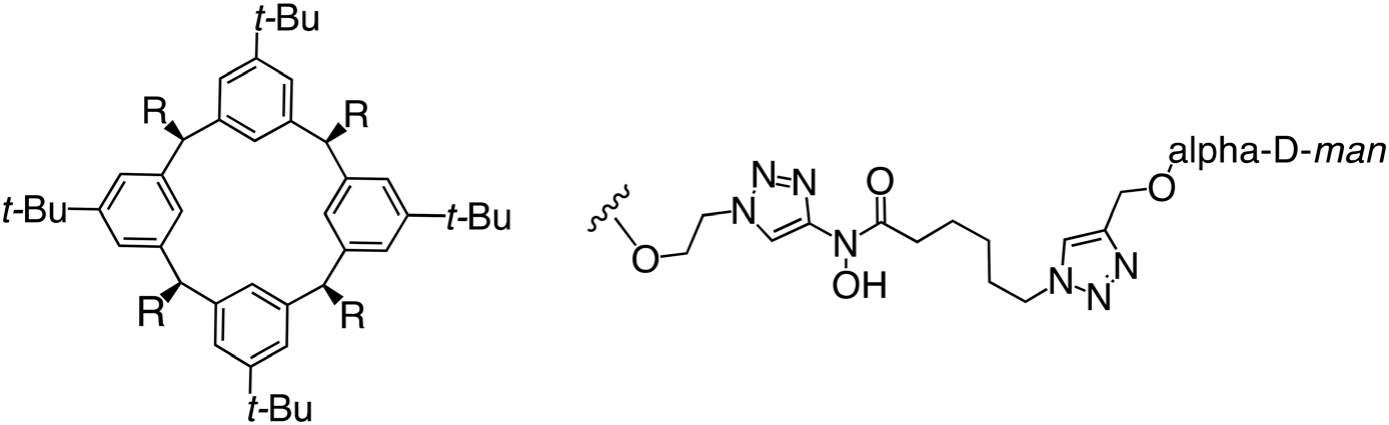	70
**Glycoconjugate**1d	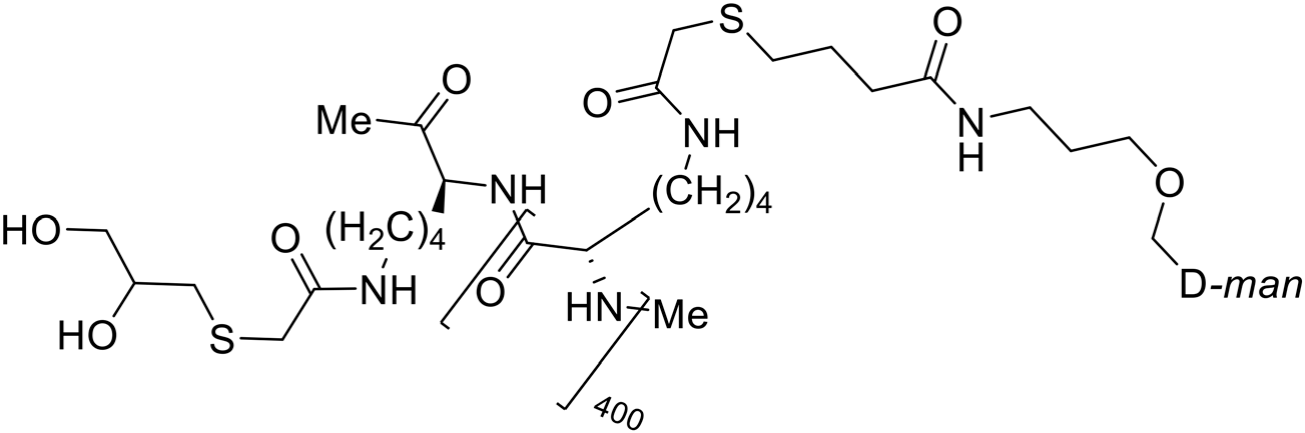	72

Because EBOV relies on promiscuous binding to glycan-recognizing surface receptors for attachment, small molecules that target host factors at the EBOV attachment step will also target other pathogens that rely on similar surface-cell receptors, including HIV, SARS-CoV-2, and some bacteria. Therefore, developing EBOV/cell attachment inhibitors could be useful for broad-spectrum anti-infective or combination therapeutics.

### Macropinocytosis

To determine the full filoviral entry mechanism, researchers have studied how EBOV enters the host cell. EBOV does not use clathrin-, caveolae-, or dynamin-dependent uptake. Instead, EBOV is macropinocytosed into the host cell and trafficked to the endososomal pathway. This mechanism was elucidated in part by use of ethylisopropylamiloride (**EIPA**), a known macropinocytosis inhibitor, to reduce infectious EBOV entry in Vero cells (Table 2).^26^ Use of infectious virus was essential for this early discovery stage to ensure clinical relevance. Similar results were recapitulated with **EIPA**'s dose-dependent inhibition of VSV-EBOV GP and VLPs in Vero cells. Other macropinocytosis inhibitors, including **LatA**, an actin polymerization inhibitor; **Rottlerin**, a protein kinase C (PKC) inhibitor; and **ML9**, a light chain kinase inhibitor, also blocked VSV-EBOV GP infection in Vero cells, dendritic cells, and peripheral blood-derived monocytes.^73^

**Table 2:** Names, structures, and references of EBOV entry inhibitors that target macropinocytosis

Name	Structure	Ref
**EIPA**	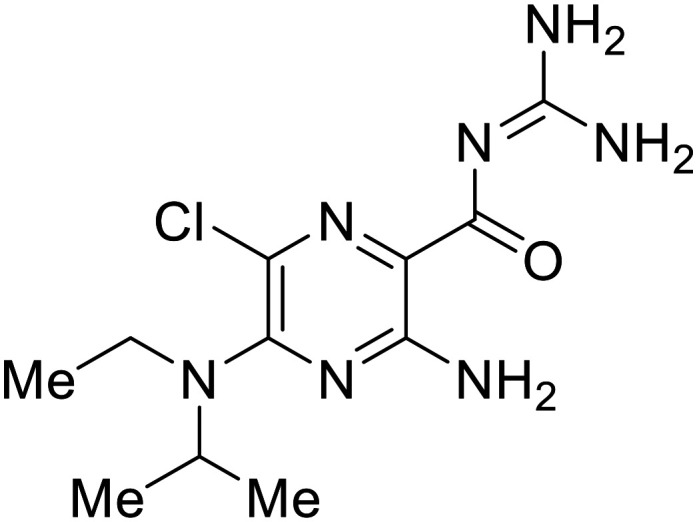	26
**LatA**	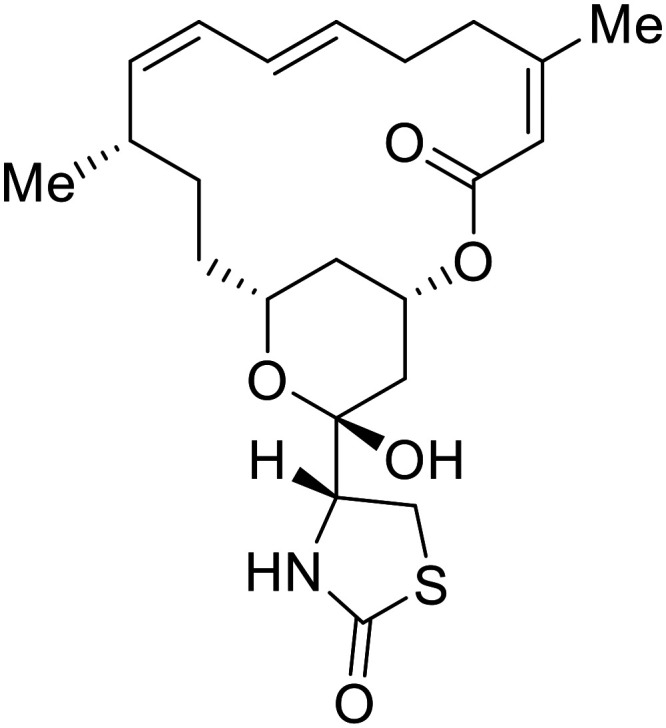	73
**Rotterlin**	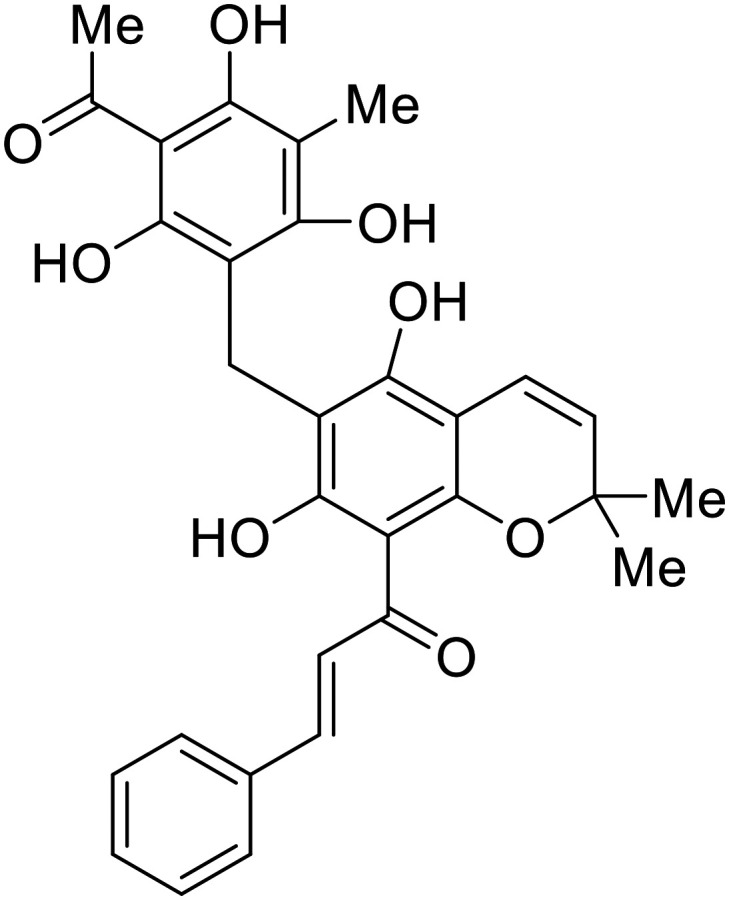	73
**ML9**	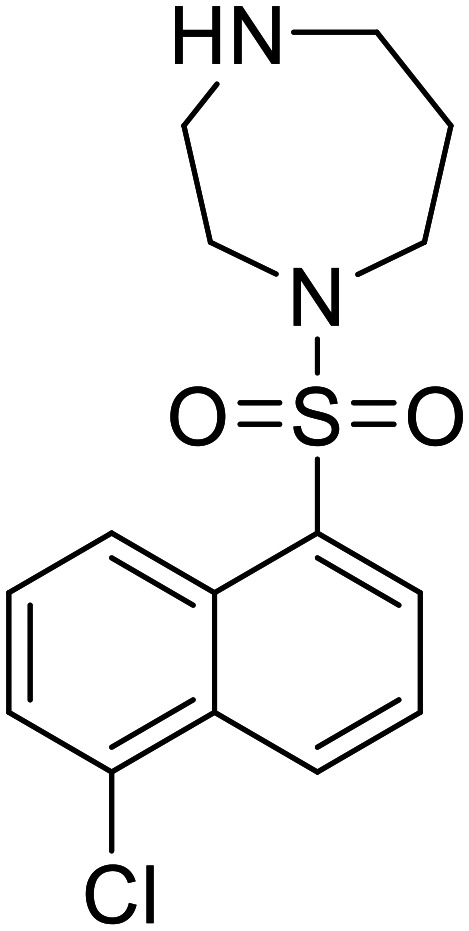	73
**R-59-022**	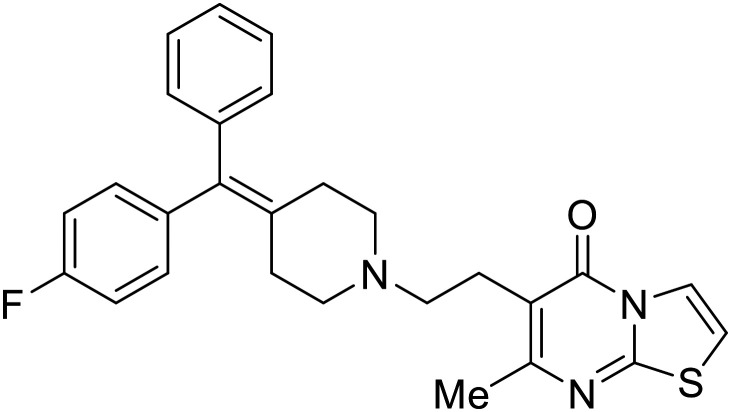	74

More recent studies discovered diacyl glycerol kinase (DGK) antagonist **R-59-022** as a filoviral entry inhibitor.^74^**R-59-022** reduced pseudotyped EBOV entry in Vero cells, as well as VLP entry in Vero and bone marrow-derived macrophages. Time-of-addition studies with **R-59-022** showcased EBOV inhibition at an earlier time point compared to NH_4_Cl, an endolysosomal pH neutralizer that prevents pH-dependent EBOV GP proteolysis. Complete viral entry inhibition within one hour supports use of **R-59-022** as a macropinocytosis inhibitor for filoviral entry. **R-59-022** is more potent (5 μM) than **EIPA** (30 μM) and provides a useful starting point and scaffold to develop more potent macropinocytosis inhibitors for EBOV.

### Proteolytic cleavage

EBOV GP_1_ contains a mucin-like domain and glycan cap. Chandran *et al.* found that low pH-dependent proteases Cathepsin L (CatL) and Cathepsin B (CatB) were required for entry, as these proteases remove the GP_1_ glycan cap that allows for GP-receptor binding in the later steps of entry.^27^ For this discovery, CatB inhibitor **CA074** and CatB/L inhibitor **FYdmk** (Table 3) were found to dose-dependently interrupt VSV-EBOV GPΔMuc and infectious EBOV entry in Vero cells. Derivative **CA074Me** was also effective in blocking VSV-EBOV GP and HIV-EBOV GP entry.^75,76^ Although these studies provided proof of concept for filoviral therapeutic development, **CA074** and its derivatives are non-ideal clinical candidates, as EBOV resistance arose within two VSV-EBOV GPΔMuc passages in **CA074**-treated Vero cells.^77^

**Table 3:** Names, structures, and references of EBOV entry inhibitors that target GP proteolytic cleavage

Name	Structure	Ref
**CA074**	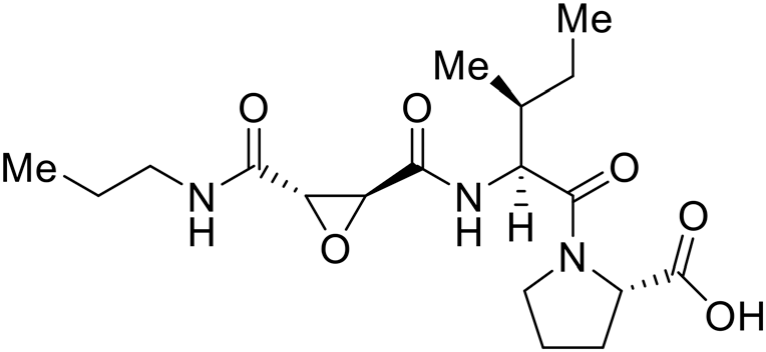	27
**FYdmk**	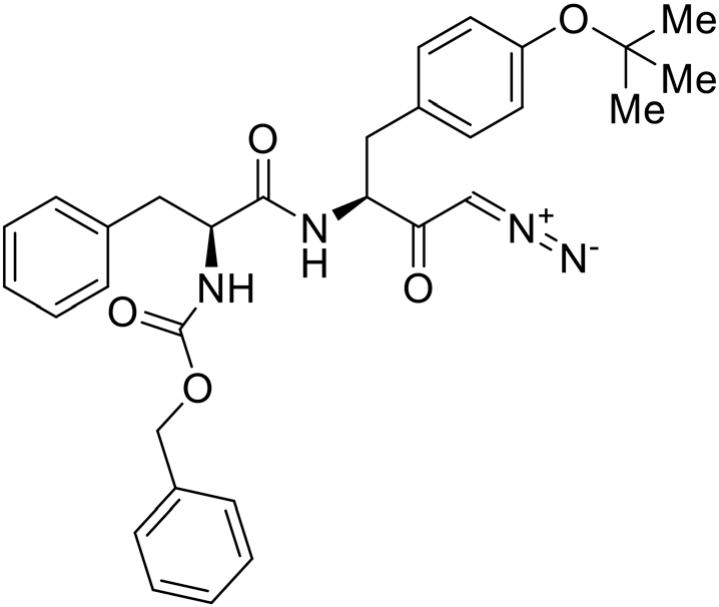	27
**CA074Me**	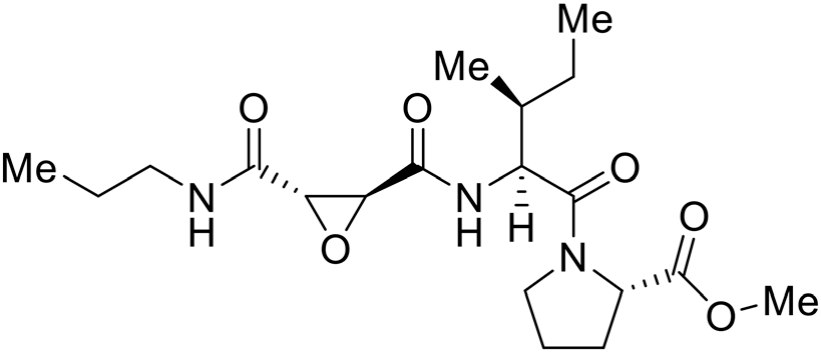	75–77
**MDL28170**	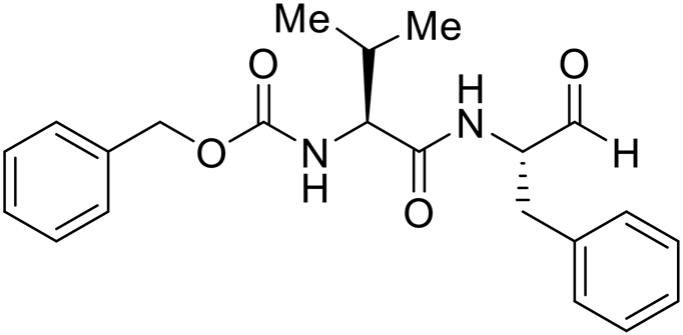	76, 78, 79
**E64D**	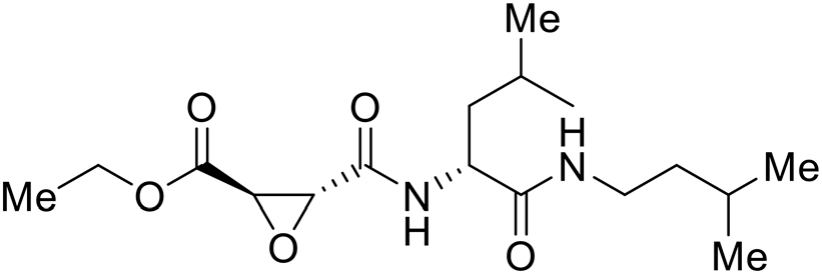	27, 76
**AMS36**	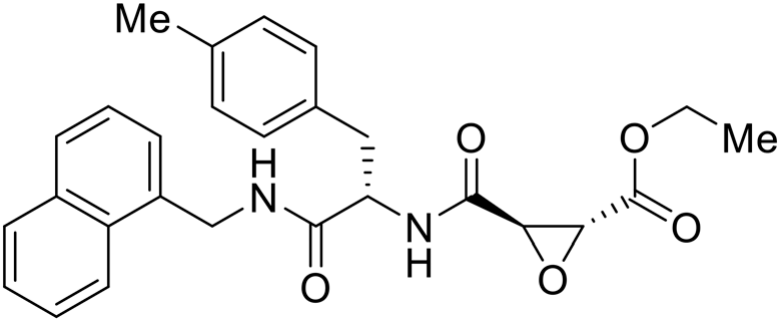	80
**R11Et**	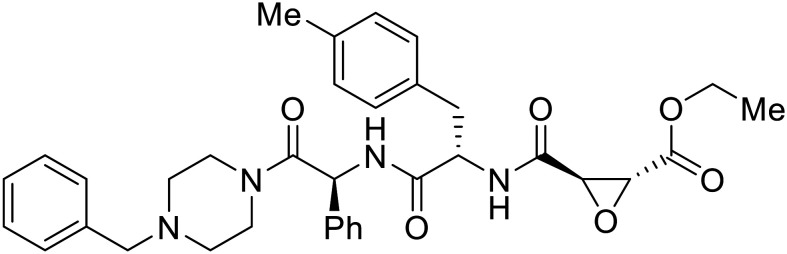	80
**R11P**	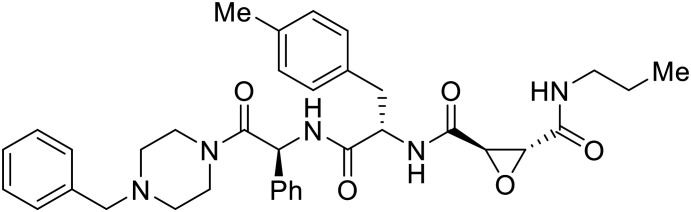	80
**K11777**	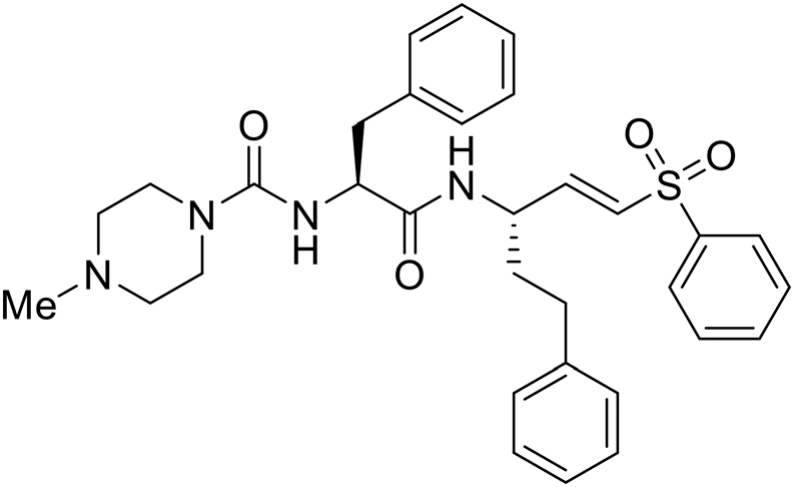	81
**PF429242**	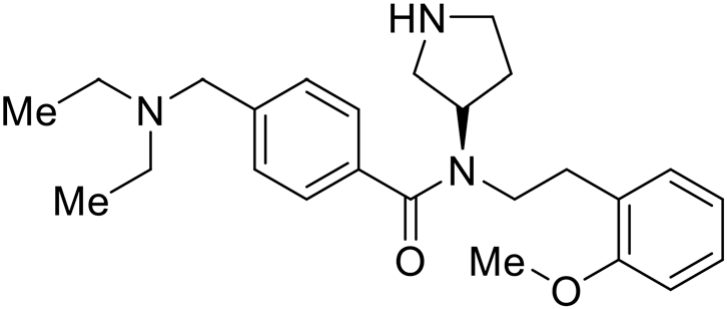	82
**(Z-LL)**_**2**_ **-ketone**	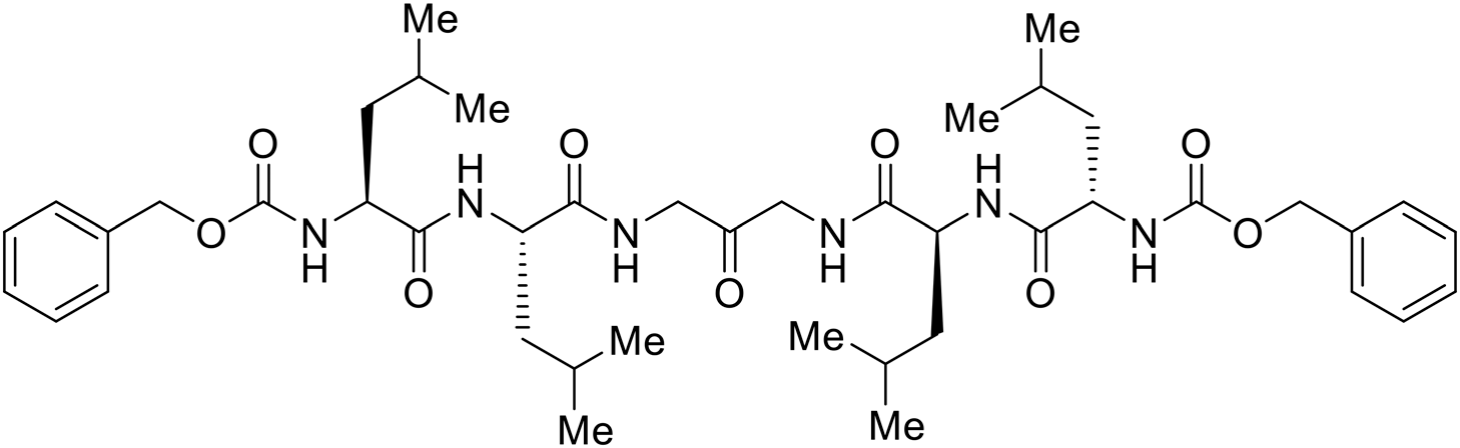	82
**Aloperine**	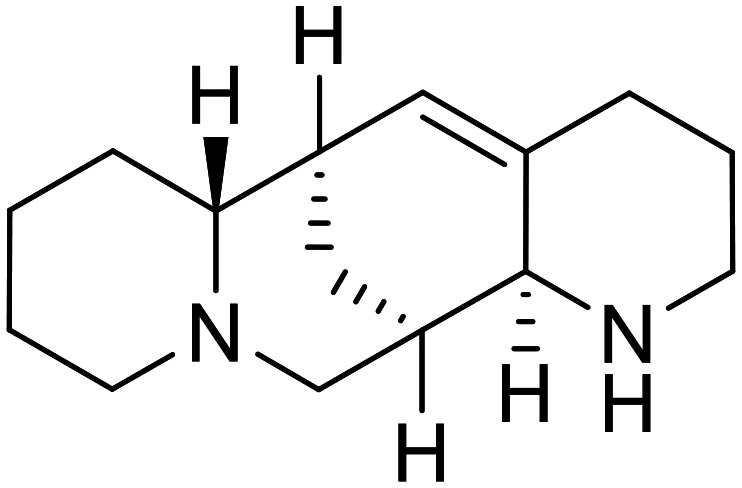	83, 84
2e	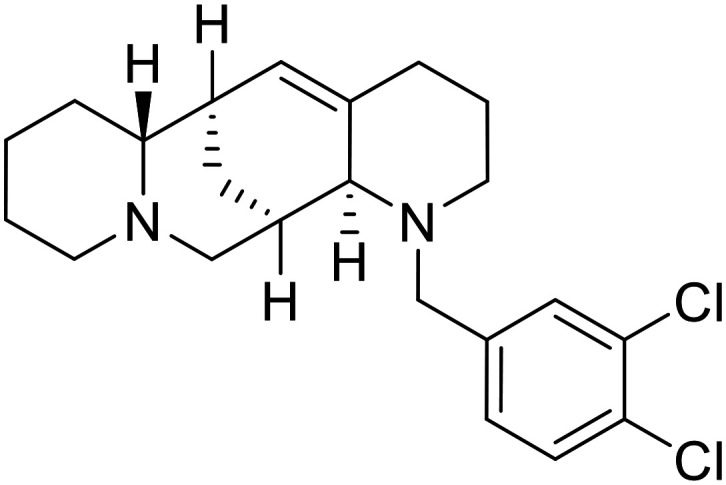	84

A similar trajectory occurred for **MDL28170**, a cysteine protease inhibitor.^78^ Cellular pretreatment with **MDL28170** at 0.5 and 10 μM effectively inhibited HIV-EBOV GP entry in 293 T cells;^76^ however, later studies determined its resistance using replication-competent VSV-EBOV GP.^79^ In a total of five passages, V37A and S195R mutations developed. GP_1_ mutation V37A was more aggressive as it occurred within the first passage and contributed more to EBOV resistance to protease inhibitors.

Broad cysteine protease inhibitor **E64D** has also effectively attenuated EBOV entry;^27,76^ however, poor permeability of this epoxide-based inhibitor required high doses of up to 300 μM to exert pharmacological effect. Maintaining the use of epoxide-based small molecules, **AMS36** was used as a scaffold to develop new cathepsin inhibitors for reduced EBOV entry.^80^ Various amines containing an aliphatic or aromatic group were coupled to the **AMS36** scaffold, while the epoxide stereochemistry was varied. Inhibitors containing an *R*,*R*-epoxide motif and a basic amine were most potent, as basic functionality is expected to assist in directing the therapeutic agents to the endolysosomal sites of filoviral entry. Rounds of SAR development of the potent derivative **R11Et** generated **R11P** that replaced **R11Et**'s labile ethyl ester with a propylamide. The amide modification improved serum stability and achieved nanomolar entry inhibition against VSV-EBOV GP (EC_50_ = 1.2 nM) and infectious EBOV (EC_50_ = 70 nM) in U2OS cells.

SAR development for cysteine protease antagonist **K11777** helped establish a pan-filoviral entry inhibitor.^81^**K11777** with sub-nanomolar activity against HIV-EBOV Zaire GP (EC_50_ = 0.87 nM) displayed additional nanomolar inhibition against pseudotyped SUDV (EC_50_ = 1.14 nM), TAFV (EC_50_ = 2.26 nM), RESTV (EC_50_ = 3.37 nM), BDBV (EC_50_ = 5.91 nM), and MARV (EC_50_ = 1.90 nM). Replacement of the 1-methyl piperazine with a 1-cyclopropylmethyl, *t*-Bu, or ethyl piperazine improved Zaire activity 8.7, 7.9, and 7.3-fold, respectively.

Cysteine protease inhibitor **(Z-LL)**_**2**_**-ketone** inactivated both CatB and CatL as expected; however, serine protease inhibitor **PF429242** was surprisingly active against cysteine protease CatB.^82^ Instead of inhibiting protease activity, **PF429242** blocked CatB-endolysosomal localization for inactivation. Both **(Z-LL)**_**2**_**-ketone** and **PF429242** caused dose-dependent entry inhibition of VSV-EBOV GP at early time points, with additional activity against pseudotyped SUDV, TAFV, BDBV, and MARV.

Natural products have also served as starting points for therapeutics targeting the GP proteolytic step. **Aloperine**, extracted from the seeds and leaves of Chinese plant *Sophora alopecuroides* L.,^83^ displayed activity against HIV-EBOV GP in HEK-293 T cells.^84^ SAR exploration *via N*-alkylation, −acylation, and -sulfonylation generated derivative 2e containing an *N*-coupled 3′,4′-dichlorophenyl group. EBOV entry inhibition of 2e improved 2.6-fold (EC_50_ = 4.8 μM) compared to **aloperine** and was effective in reducing the presence of HIV-EBOV GP virus in treated BALB/c mice compared to untreated mice. These *in vivo* studies exemplify the use of protease inhibitors in more complex systems; however, further studies using clinically relevant infectious virus are needed.

### NPC1 binding

In 2011, researchers found that proteolysis mediated by pH-dependent cathepsins reveals the receptor binding site (RBS) in GP_1_ that enables the GP-NPC1 binding event required for EBOV entry.^85^ The screening of a small-molecule library against VSV-EBOV GP identified benzylpiperazine adamantane **3.0** as an entry inhibitor. Initial SAR development generated derivative **3.47** with sub-micromolar EBOV inhibition (Table 4). Both compounds induced cholesterol-accumulation in endolysosomal compartments, helping to identify NPC1 as their target. Immunoprecipitation assays revealed binding between proteolytically cleaved GP_1_ and NPC1, which was inhibited by dose-dependent administration of **3.0** and **3.47**. Though potent, **3.47** contains an adamantane group contributing to an increased log *P* (7.2) and a labile methyl ester that compromises metabolic stability. Replacement of the adamantane with a difluoro spiro[2.5]octane, and methyl ester with a methyl sulfone generated derivatives 3–22 and 3–25 that improved infectious EBOV entry inhibition from 64 nM, to 19 and 21 nM, respectively.^86^

**Table 4:** Names, structures, and references of EBOV entry inhibitors that target the GP-NPC1 binding step

Name	Structure	Ref
**3.0**	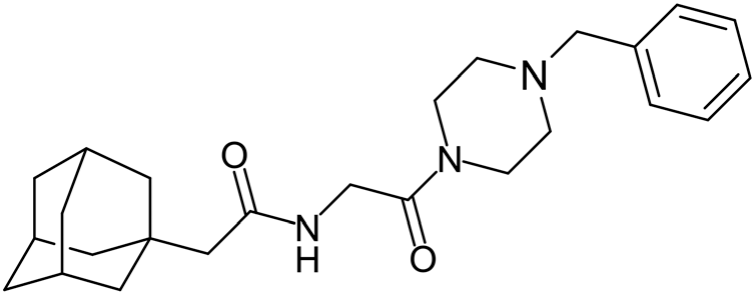	85
**3.47**	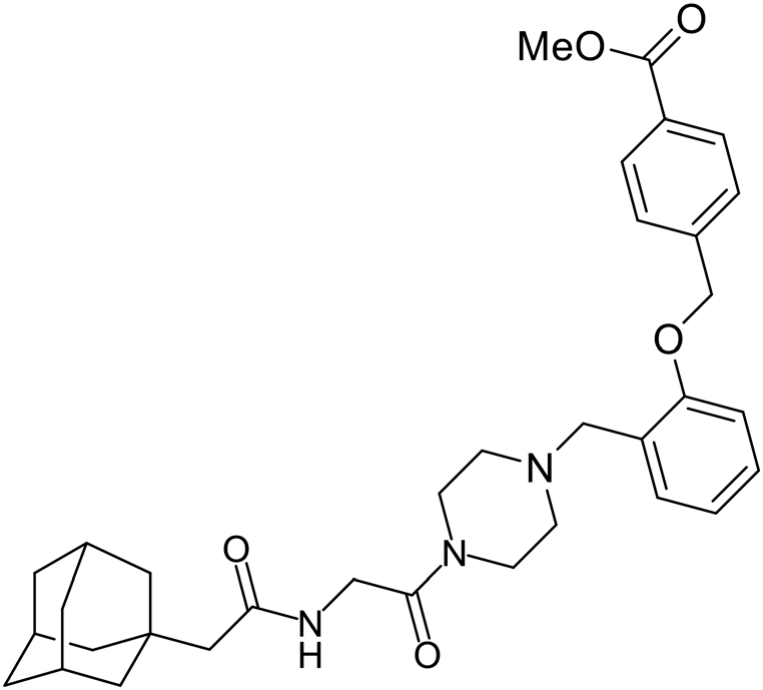	85
3–22	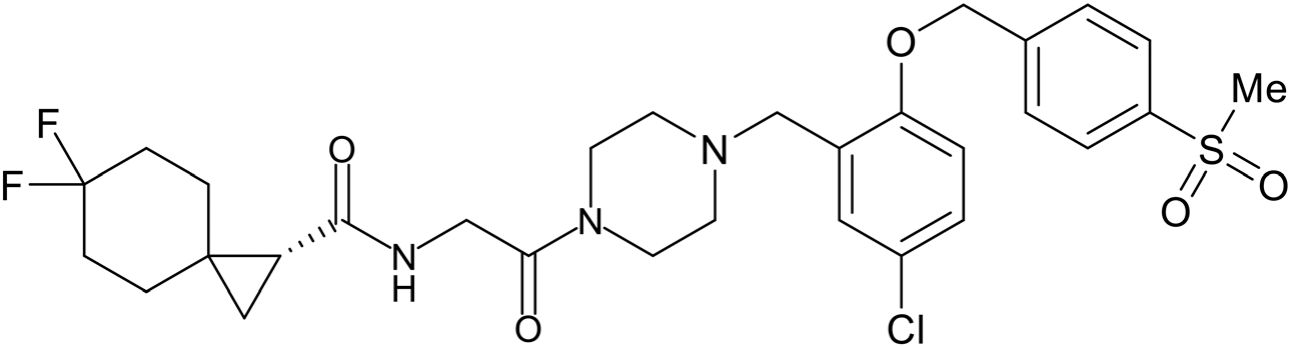	86
3–25	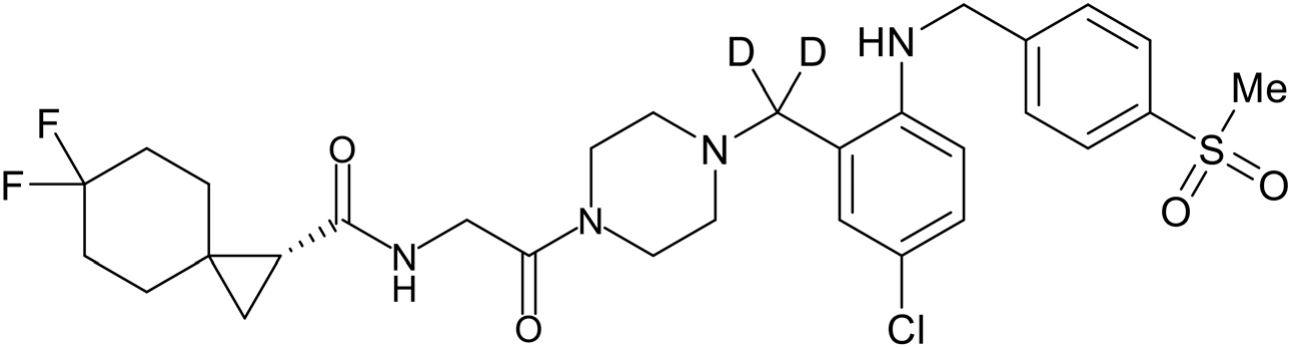	86
**MBX2254**	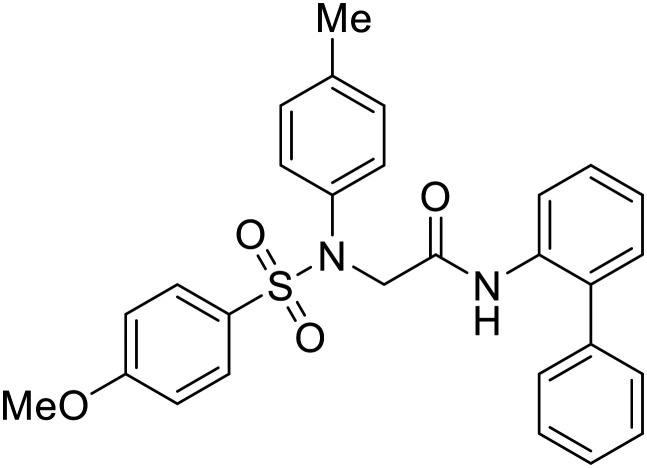	87
**MBX2270**	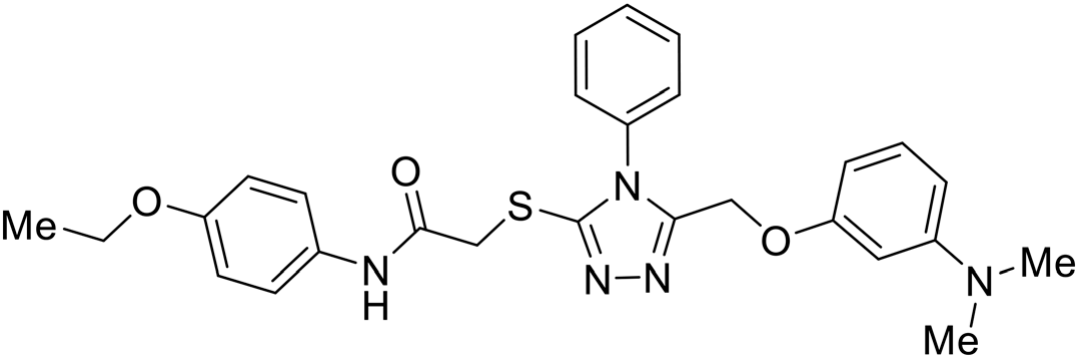	87
**Tubeimoside I**	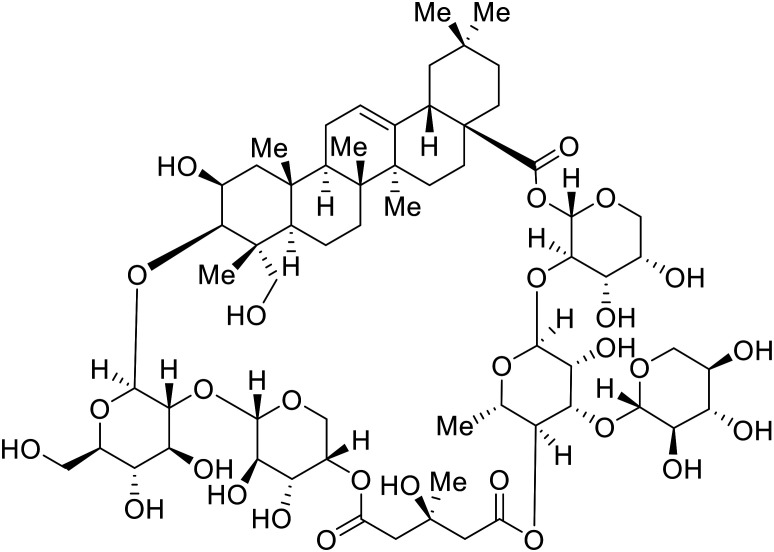	88
**Tubeimoside II**	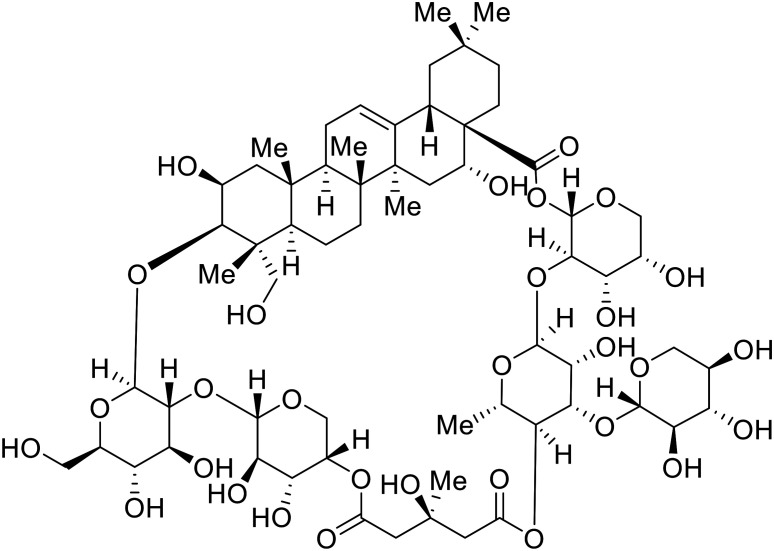	88
**Tubeimoside III**	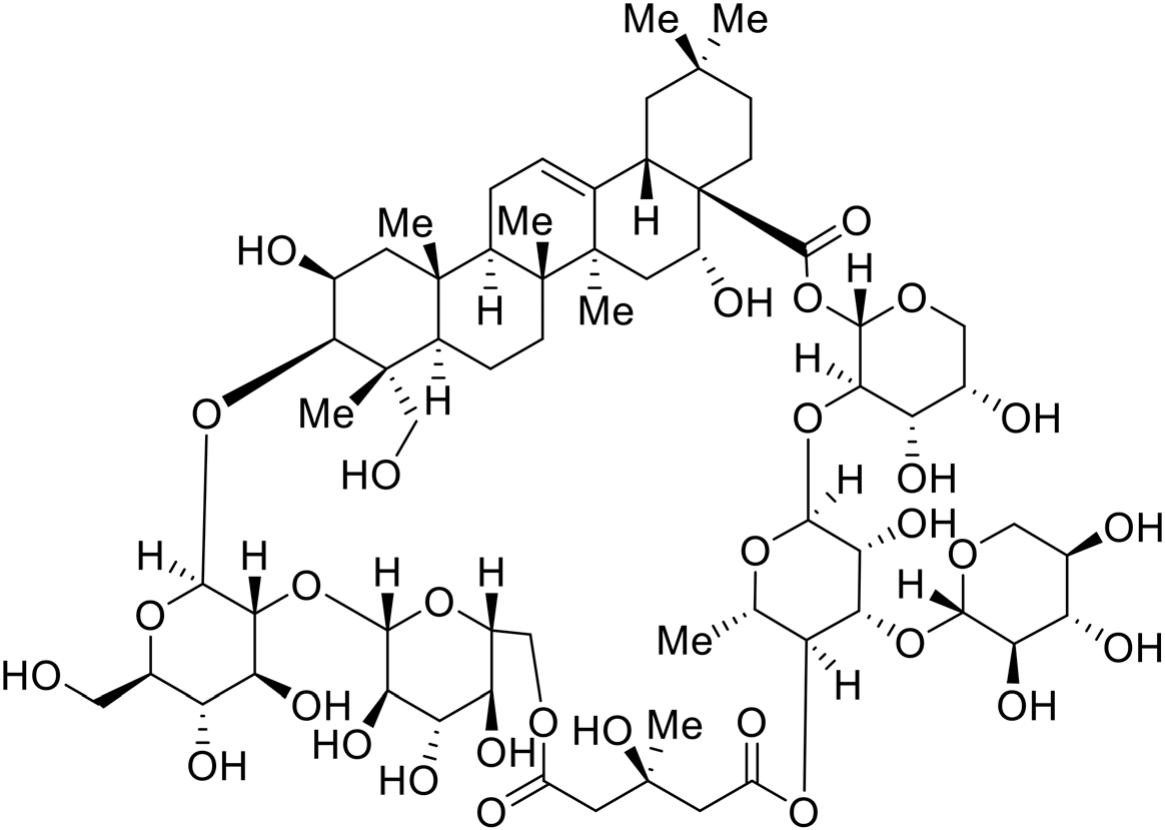	88
**Itraconazole**	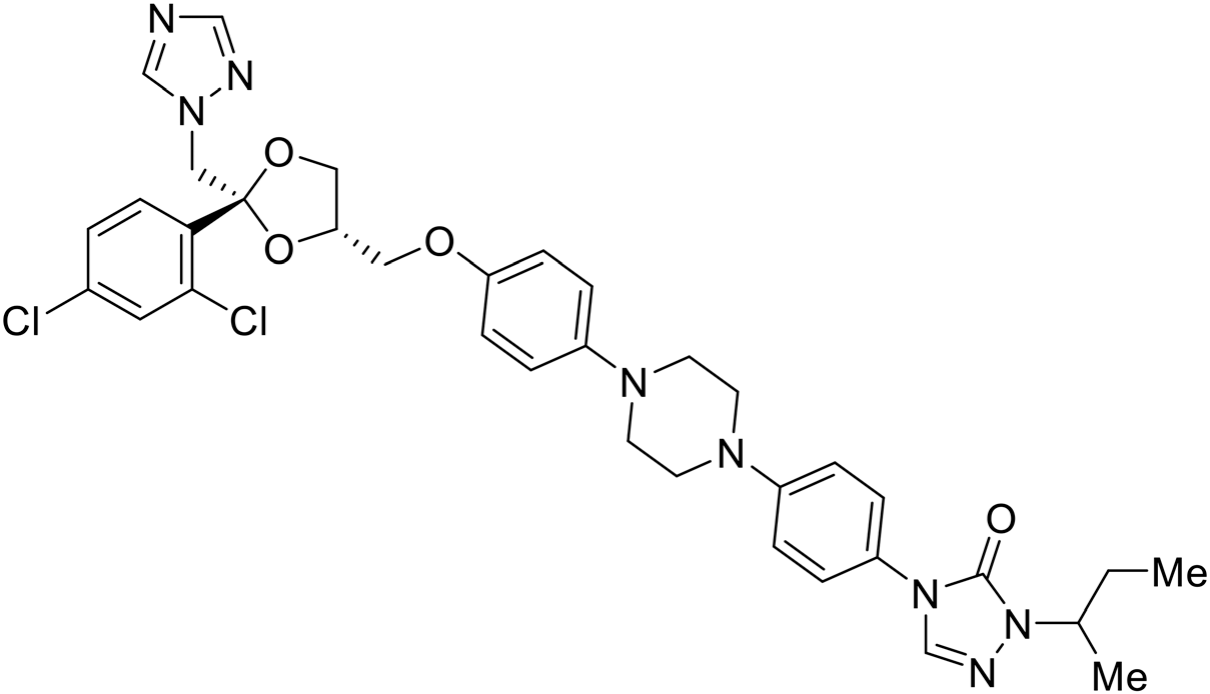	89, 90
**Berbamine hydrochloride**	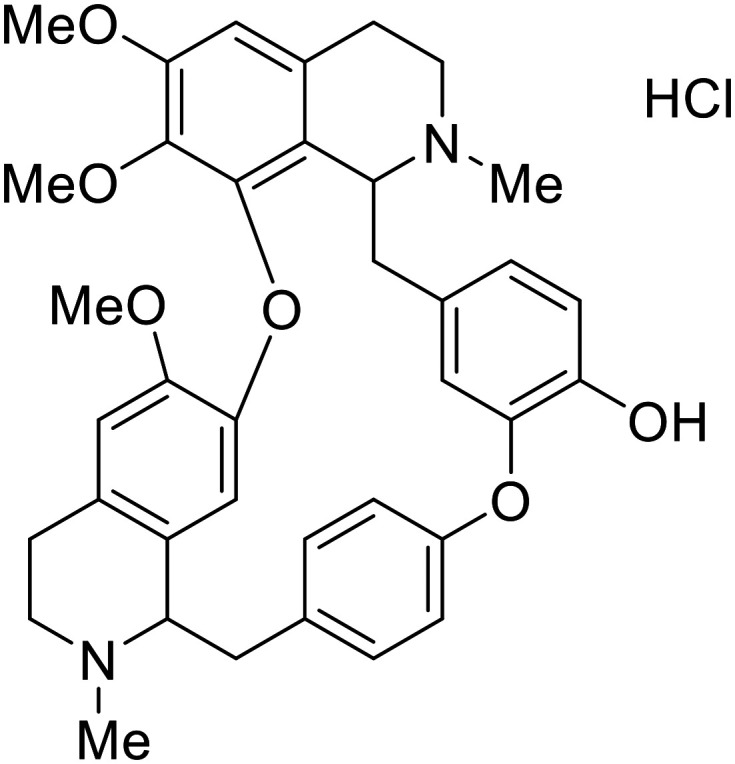	91
**U18666A**	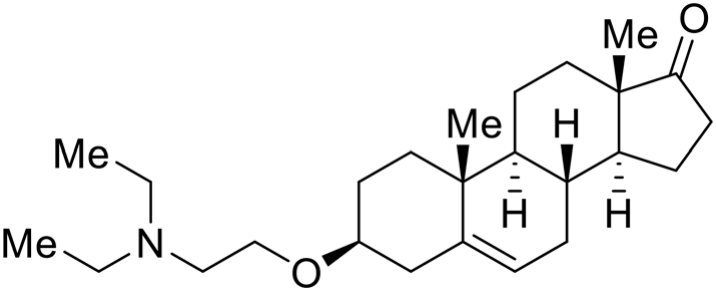	85, 92, 93, 95
**Compound**9	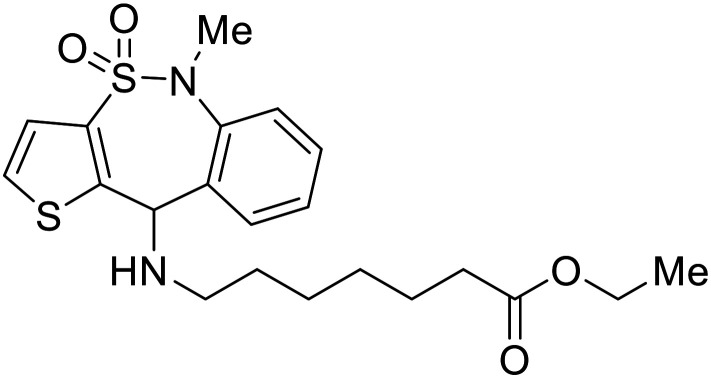	95
**Lamellarinα sulfate**	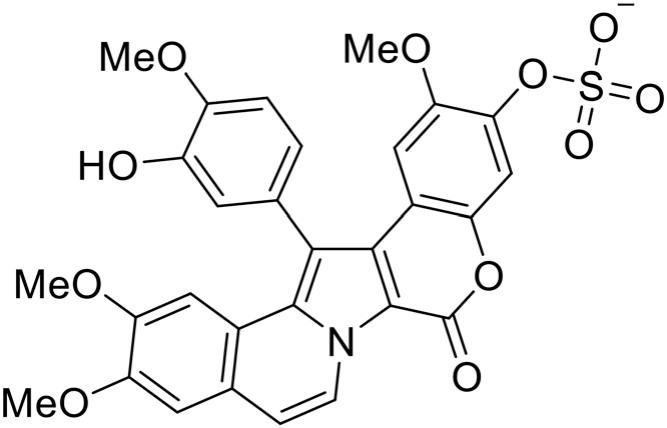	96
A	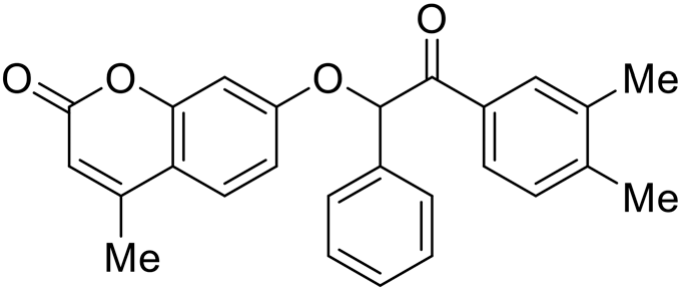	97
U	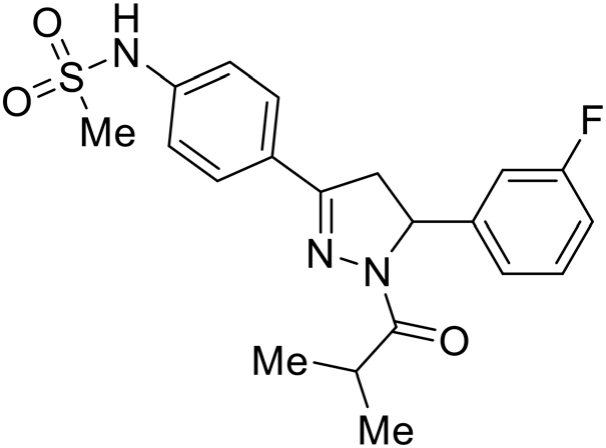	97

Identification of NPC1 as an integral protein in EBOV entry led to the pursuit of small molecules that inhibit this essential binding interaction. A small-molecule high-throughput screen identified **MBX2270** and **MBX2254** as HIV-EBOV GP entry inhibitors.^87^ Endolysosomal pH was not altered by **MBX2270** nor **MBX2254**; however, cholesterol accumulation was induced in A549 cells. Both **MBX2270** and **MBX2254** additionally blocked the NPC1-GP_1_ binding event as determined by an AlphaLISA experiment. Potent **tubeimosides I**, **II**, and **III** derived from *Bolbostemma paniculatum* inhibited HIV-EBOV GP entry in Vero and SNB-19 cells, and EBOV transcription and replication-competent VLP's in HEK-293 T cells (EC_50_ < 200 nM).^88^ Docking models suggest that **tubeimosides I**, **II**, and **III** block NPC1's loop 1 from protruding into the EBOV RBS, disrupting the NPC1-EBOV GP_1_ binding interaction.

Known NPC1 binder **itraconazole**^89^ demonstrated infectious EBOV entry inhibition in MoKi, Vero E6, and A549 cells.^90^ Bio-layer interferometry (BLI) and pull-down assays determined *Berberis amurensis* natural product **berbamine hydrochloride** as a thermolysin-treated GP_1_ binder.^91^ When administered to BALB/c mice either 1 day pre- or post- mouse-adapted EBOV infection, **berbamine hydrochloride** treatment resulted in 100% and 83% survival rates, respectively, with 0% survival among the control-treated animals.

**U18666A** ^92,93^ was explored due its ability to disrupt endosomal cholesterol export by binding NPC1 at the sterol-sensing domain.^94^ Both VLP and infectious EBOV entry (EC_50_ = 8.05 μM) were inhibited by its dose-dependent administration of **U18666A** in Vero cells. Similarly, benzothiazepine **compound**9 also inhibits EBOV entry, but lacks evidence for EBOV GP or NPC1 binding.^95^ Additional studies are needed to elucidate the exact mechanism of action of **compound**9, although endolysosomal cholesterol accumulation is thought to be a contributing factor.

Virtual screening of suggested NPC1/EBOV GP-targeting inhibitors has proven to be a useful tool in the identification of small molecules with more clearly defined mechanisms of entry inhibition. Natural product **lamellarin α sulfate**, a marine alkaloid with broad-antiviral activity, decreased HIV-EBOV GP infection in HEK-293 T cells in a dose-dependent manner, albeit at high concentrations (50, 100, and 150 μM).^96^ Increased doses are needed for this natural product due to its membrane impermeability, attributed to the negatively charged sulfate. Additionally, A (chrome-2-one-based) and U (pyrazole-based) compounds were identified as potential EBOV GP_1_ binders at the RBS region.^97^ Moderate HIV-EBOV GP entry inhibition for the initial A and U compounds, 11.9 and 8.77 μM respectively, led to the SAR exploration of each. Unfortunately, chemical modifications to each scaffold either compromised cytotoxicity or lacked potency improvements. Additional SAR developments of **lamellarin α sulfate**, A, and U compounds are needed, along with binding confirmation to their indicated targets.

### Fusion

During the early stages of the largest EBOV epidemic in 2013, the need for effective EBOV therapeutics was re-emphasized. An FDA-approved small molecule library was screened against EBOV to identify drugs with repurposing potential.^98^ Many of the drugs screened were selective estrogen receptor modulators (SERMs), with **toremifene** (EC_50_ = 0.162 μM) and **clomiphene** (EC_50_ = 2.42 μM) being the most potent against infectious EBOV in Vero cells (Table 5). These SERMs were of interest because they maintained their antifiloviral activity despite the lack of cellular ERα presence, which suggested a distinct antiviral mechanism. Continued studies led to the co-crystallization of **toremifene** with EBOV GP, identifying the internal fusion loop region as a novel binding site for small molecules.^99^ Displacement of the GP_1_ DFF lid by **toremifene** binding was proposed to destabilize the GP conformation needed for fusion to occur. Reduction of the GP melting temperature in the presence of **toremifene** also supports this notion. Strong antifiloviral activity of SERMs led to further SERM exploration for EBOV entry inhibitors. The screening of ER ligands identified **ridaifen-B** as a candidate.^100^ Rounds of SAR and reverse engineering were used to optimize filoviral potency and reduce ER activity of **ridaifen-B**. Phenol replacement with an ethyl-linked pyrrolidine (analog 30) improved infectious EBOV (EC_50_ = 0.64 μM) and MARV entry inhibition, and reduced ER activation compared to **ridaifen-B**. Future SAR studies of **ridaifen-B** are needed to improve the cytotoxicity and further reduce ER activation.

**Table 5:** Names, structures, and references of EBOV entry inhibitors that target fusion

Name	Structure	Ref
**Toremifene**	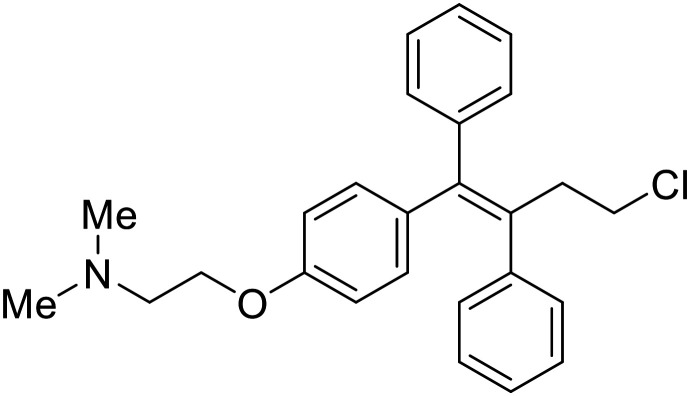	98, 99
**Clomiphene**	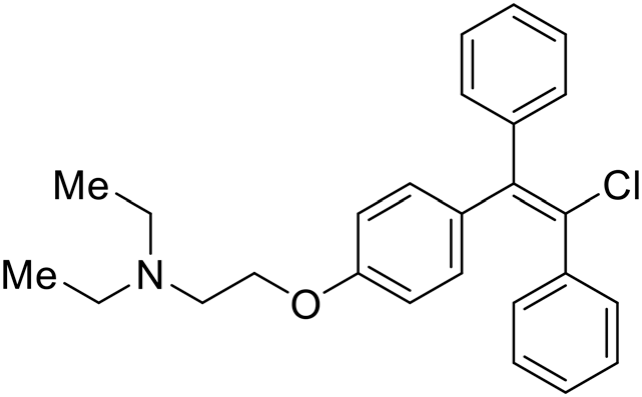	98
**Ridaifen-B**	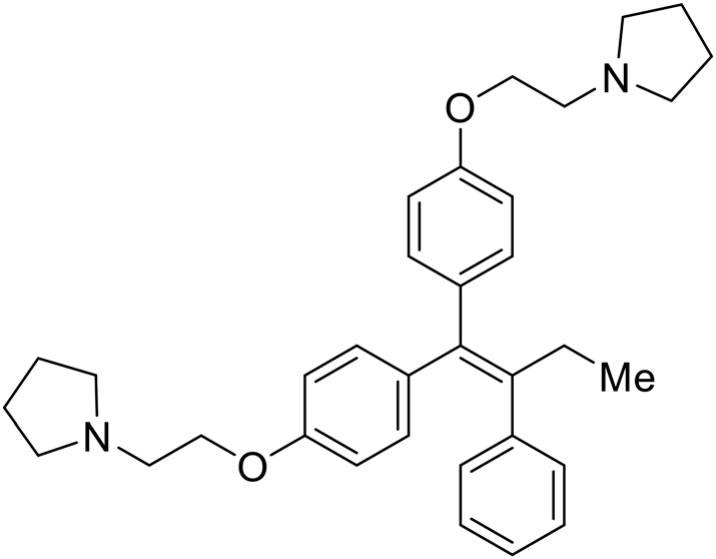	100
30	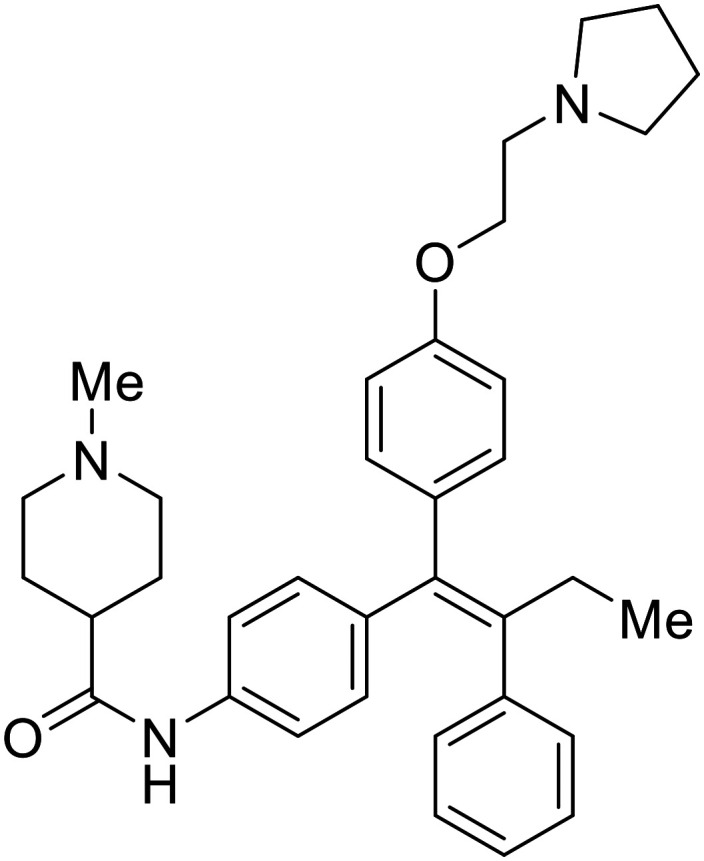	100
**Imipramine**	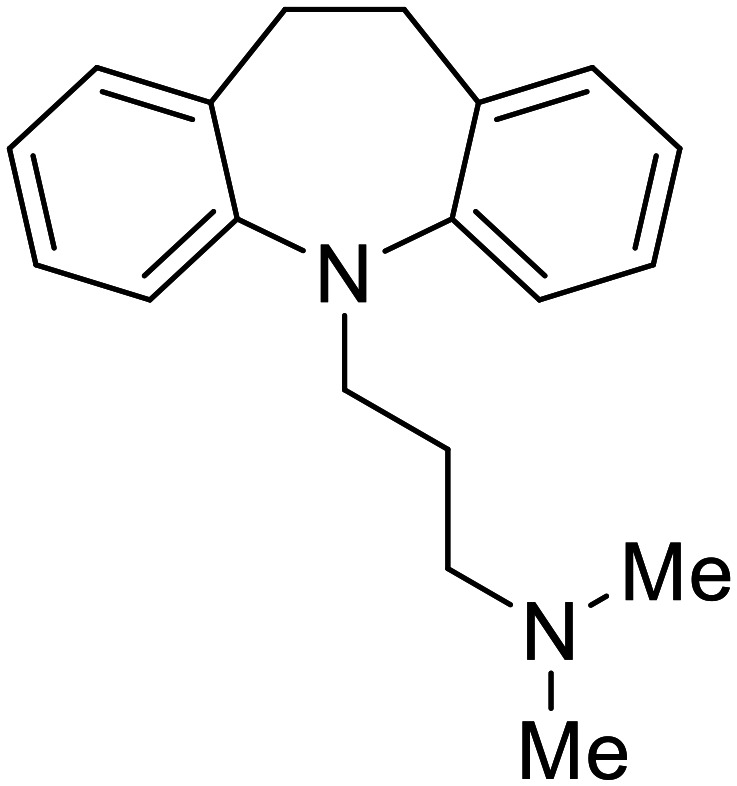	101
**Clomipramine**	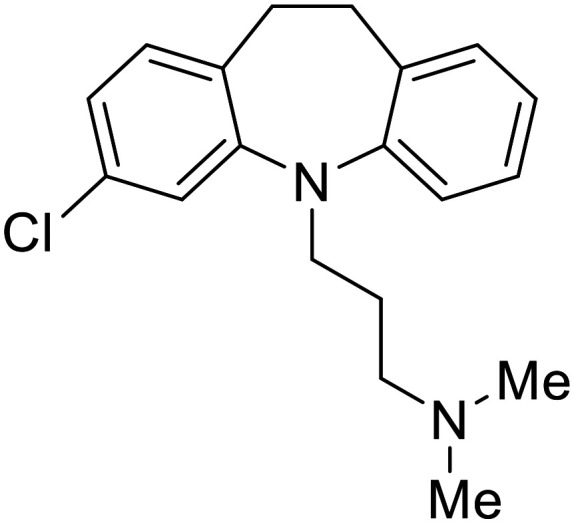	101
**Thioridazine**	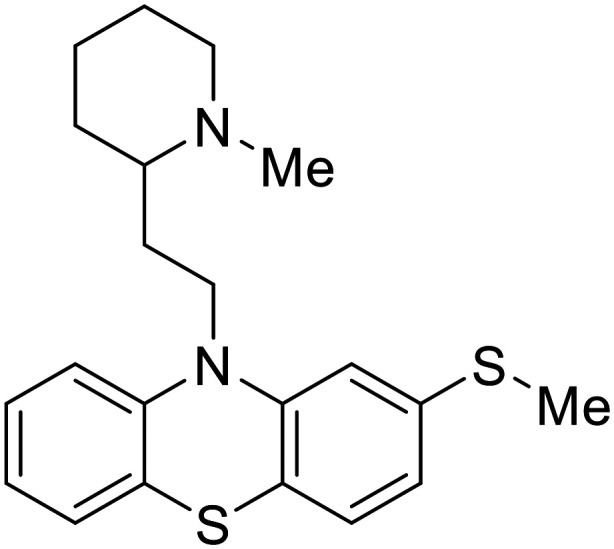	101
**118**	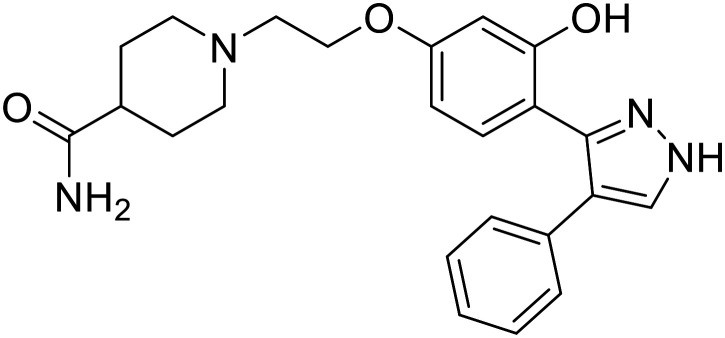	102
**118a**	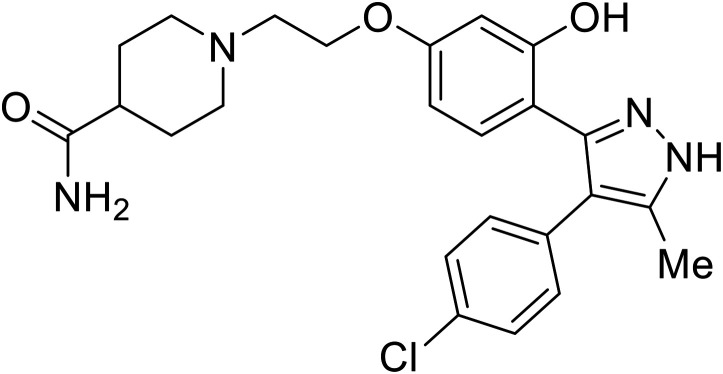	102
**Procyanidin B2**	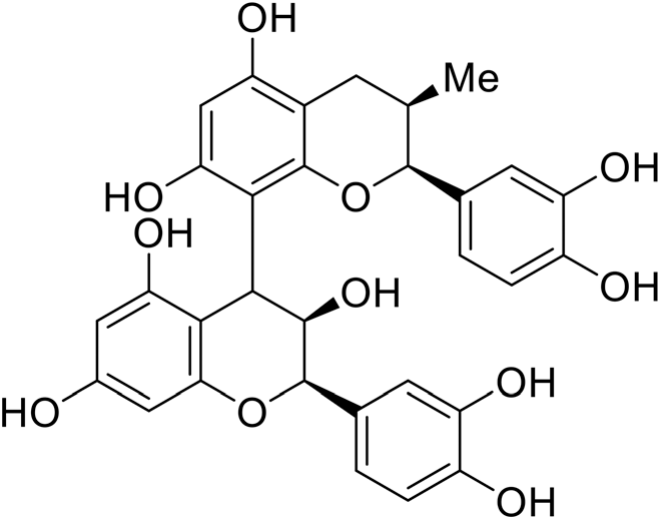	103
**Chlorcyclizine**	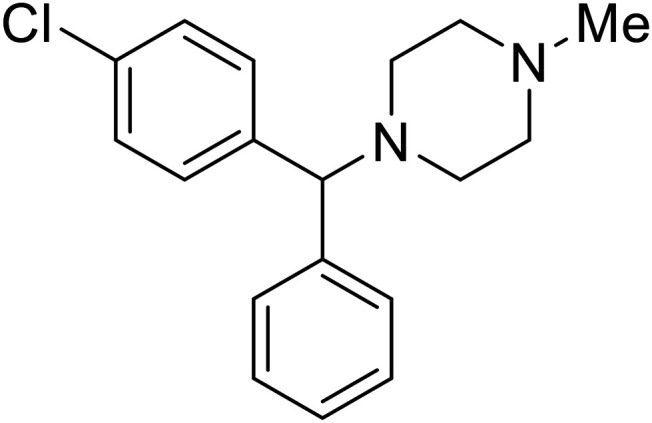	104, 105
**Diphenhydramine**	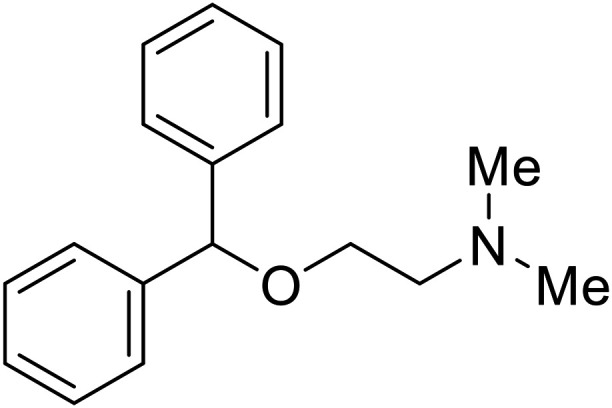	104, 105
**CP19**	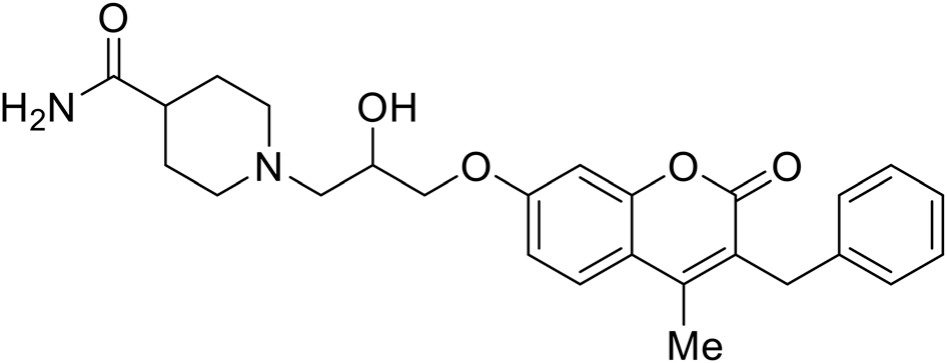	104, 105
**Tilorone**	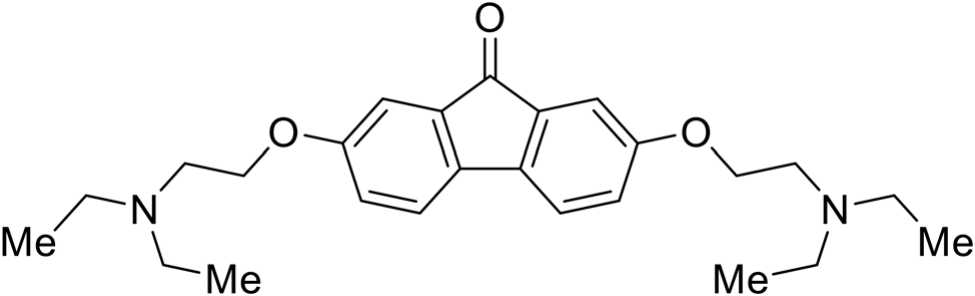	106–108
58	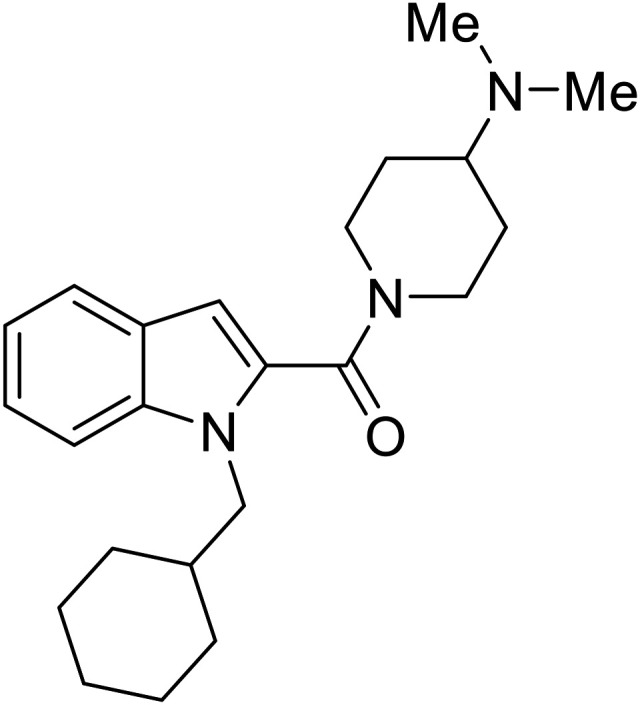	44
28	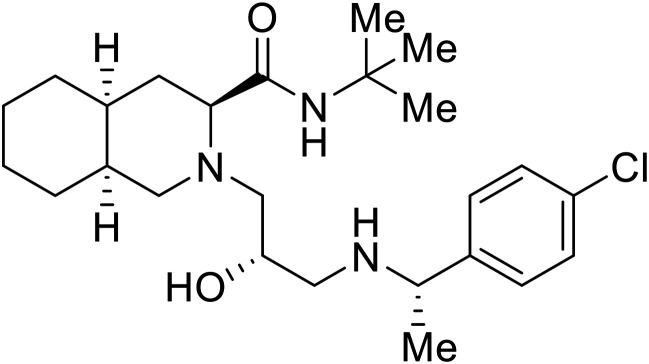	109
**SYL1712**	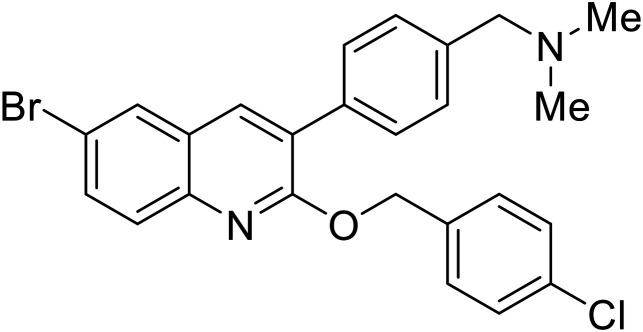	110
60	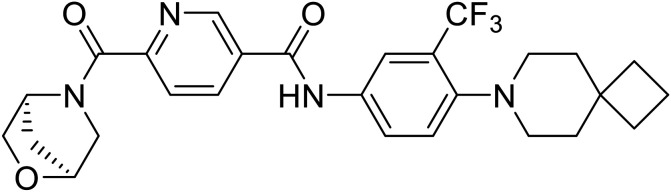	111
**4-(Aminomethyl) benzamide** (35)	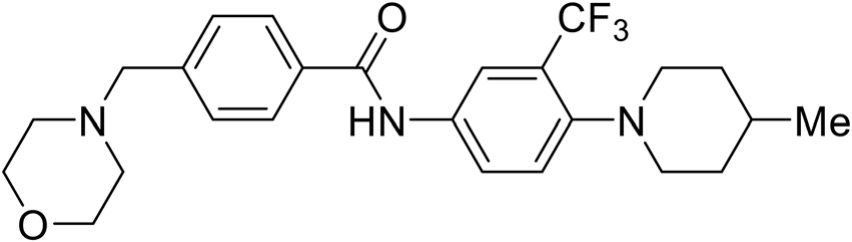	112
38	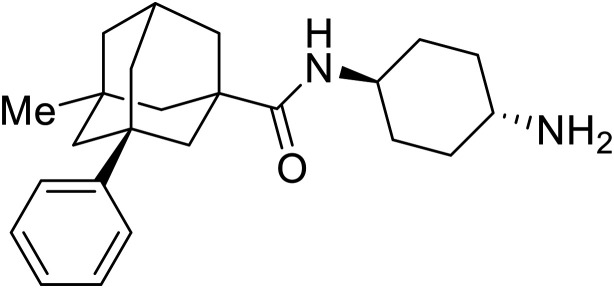	113

Other hits from the FDA-drug screen included antidepressant and antipsychotic drugs **imipramine**, **clomipramine**, and **thioridazine**. Unlike **toremifene**, **imipramine** and **clomipramine** do not destabilize GP upon increasing temperatures and lack strong binding affinity to GP (**imipramine***K*_D_ = 584 μM; **clomipramine***K*_D_ = 118 μM).^101^ Nonetheless, X-ray crystallography revealed the GP internal fusion loop region as the **imipramine-**, **clomipramine-**, and **thioridazine**-binding site with DFF lid displacement upon binding, similar to **toremifene**.

The identification of the internal fusion loop region as a confirmed GP-binding pocket enabled structure-based *in silico* screening of small molecules in this region. Traditional Chinese medicinal actives **118** (ZINC32540717) and **118a** (ZINC09410451) were identified.^102^ The co-crystal structure of **118a** (EC_50_ = 0.05 μM) bound to EBOV GP showed that two **118a** molecules occupy the internal fusion loop binding pocket, further increasing interactions with GP residues. Other traditional Chinese herbs like *Maesaperlarius* also produce filoviral entry inhibitors.^103^ The methanolic extract elucidated from this plant contains **procyanidin B2** that exhibits anti-EBOV activity and favorable cytotoxicity. Microscale thermophoresis determined **procyanidin B2**-GP binding (*K*_D_ = 13 μM), which was comparable to **toremifene** (*K*_D_ = 21 μM). Thus, natural products like **118a** and **procyanidin B2** serve as good starting points for novel entry inhibitors.

EBOV is known to compromise the immune response,^9,10^ yet antihistamines, which suppress allergic responses, inhibit filoviral entry. H1 receptor antagonists, including **chlorcyclizine**, **diphenhydramine**, and **CP19**, demonstrate moderate entry inhibition; however, H2, H3, and H4 antagonists lack activity.^104,105^ Docking and mutational analysis studies have suggested fusion inhibition as the antiviral mechanism of action of the antihistamines. Broad-spectrum antiviral **tilorone** is another approved drug that exhibits potent entry inhibition against infectious EBOV (EC_50_ = 0.23 μM).^106^ Microscale thermophoresis suggests that **tilorone** has 35-fold stronger binding to EBOV GP compared to **toremifene**.^107^ Strong binding, coupled with favorable pharmacokinetic and established safe dosing ranges of 2–10 mg kg^−1^ in mice,^108^ encourages further study of **tilorone**.

Repurposed drugs are effective starting points for the development of filoviral entry and fusion inhibitors; however, novel small molecules with fewer off-target effects are also needed. An N-substituted furopyrrole discovered in a HTS displayed activity against EBOV.^44^ Optimization of the amide-amine linker, heterocyclic core, and N-substituent generated compound 58 that maintained sub-micromolar activity in both pseudovirus and infectious EBOV assays (EC_50_ = 0.29 μM and 0.39 μM, respectively). When tested against MARV and other Ebola virus species including SUDV, BDBV, and TAFV, 58 demonstrated broad-spectrum antifiloviral activity; however, it lacked potent inhibition against non-filoviruses like influenza, showcasing selective filoviral activity. Additionally, **isoquinoline** (28),^109^**diaryl quinoline** (**SYL1712**),^110^**2,5-pyridinedicarboxamide** (60),^111^ and **4-(aminomethyl)benzamide** scaffolds^112^ were identified in separate HTS's. The **4-(aminomethyl)benzamide** derivatives seemed more promising as fusion-specific inhibitors due to the additional mutational analysis studies used. SAR of the benzamides included lipophilic adamantane coupling to the scaffold, which improved activity yet adversely increased log *P*. Fortunately, this bulky substituent proved useful for an adamantane carboxamide scaffold identified in a HTS.^113^ SAR development revealed potent inhibitors with the *S*-configuration with sub-micromolar infectious EBOV entry inhibition (EC_50_ = 0.24 μM) and favorable cytotoxicity. X-ray co-crystallography with the potent lead compound 38 and EBOV GP revealed the internal fusion loop region as the binding site. Like **toremifene** and other identified fusion inhibitors, derivative binding in this region displaced the DFF lid. Additional studies can be done to determine GP-stabilization when bound to compound 38 to suggest fusion-specific inhibition.

## Replication and transcription inhibitors

Various screens were used to identify EBOV replication and transcription inhibitors and host factors involved (Table 6), including a genome-wide siRNA screen that identified host carbamoyl-phosphate synthetase 2, aspartate transcarbamylase, and dihydroorotase (CAD) as hits.^114^ Use of a mini-genome platform found **6-azauridine** to reduce EBOV titer growth in Vero cells, as **6-azauridine** inhibits orotidylic acid decarboxylation during *de novo* pyrimidine biosynthesis.^115^ Additionally, **teriflunomide**, an FDA-approved drug that blocks the production of orotic acid from the dihydroorotic acid precursor in the uridine monophosphate synthetic pathway, inhibited *in vitro* transcription and replication in HEK293 cells. By assessing various thymidine, adenosine, cytidine, and guanosine analogs against recombinant EBOV, cytidine analog **β-d-N**^**4**^**-hydroxycytidine (NHC)** was found to inhibit EBOV genomic replication and dose-dependently attenuated EBOV infection in Vero cells and donor-derived macrophages.^116^

**Table 6:** Names, structures, and references of EBOV replication and transcription inhibitors

Name	Structure	Ref
**6-Azauridine**	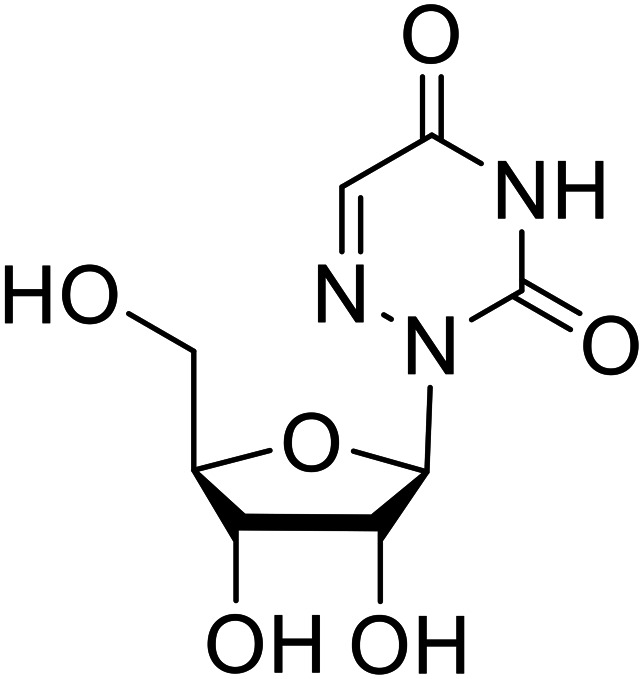	115
**Teriflunomide**	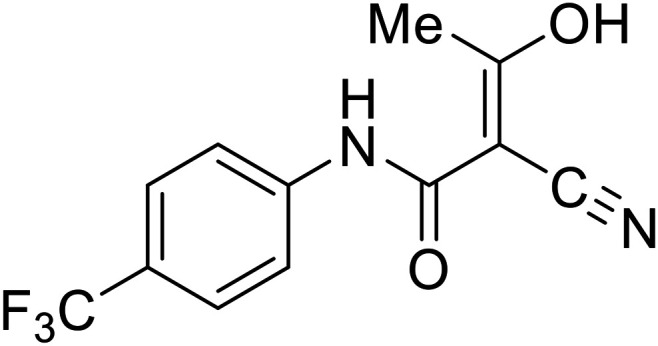	115
**β-d-N** ^**4**^ **-hydroxycytidine (NHC)**	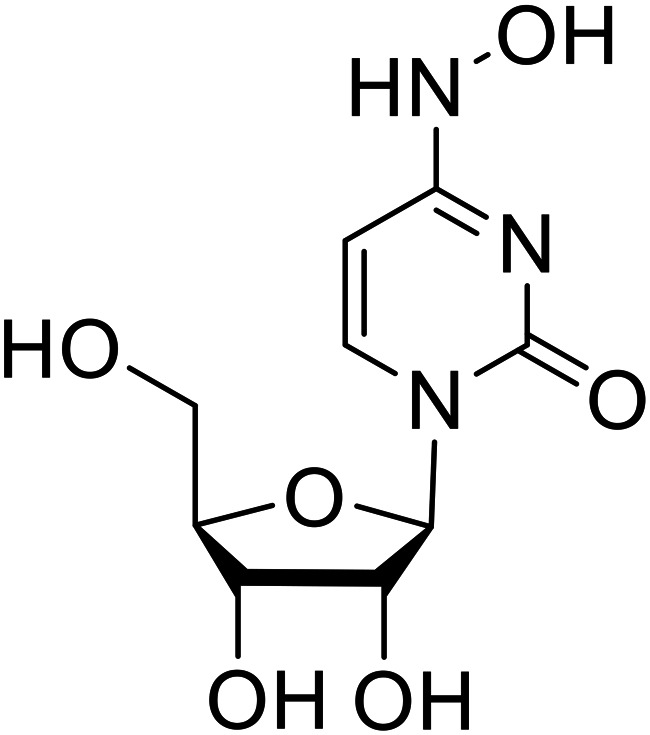	116
**Galidesivir (BCX4430)**	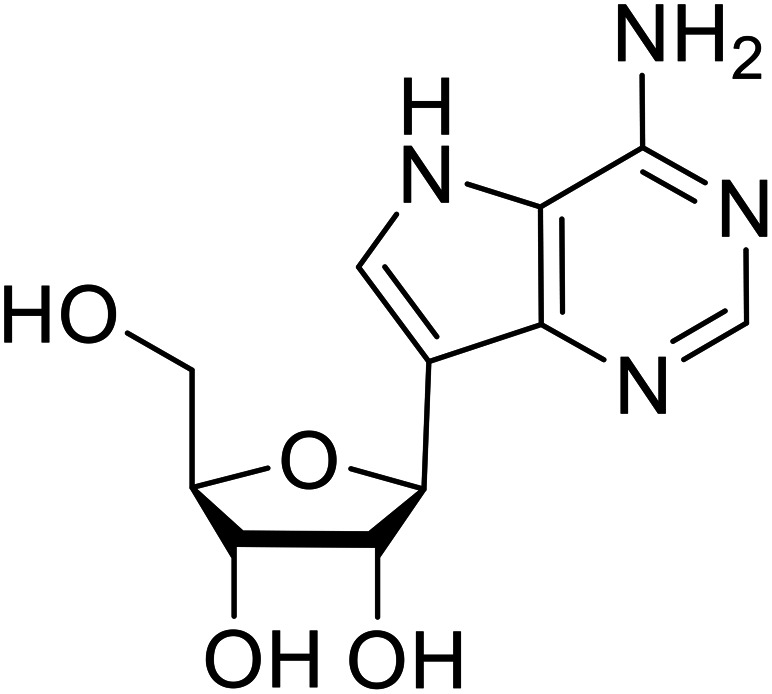	117, 118
**Favipiravir (T-705)**	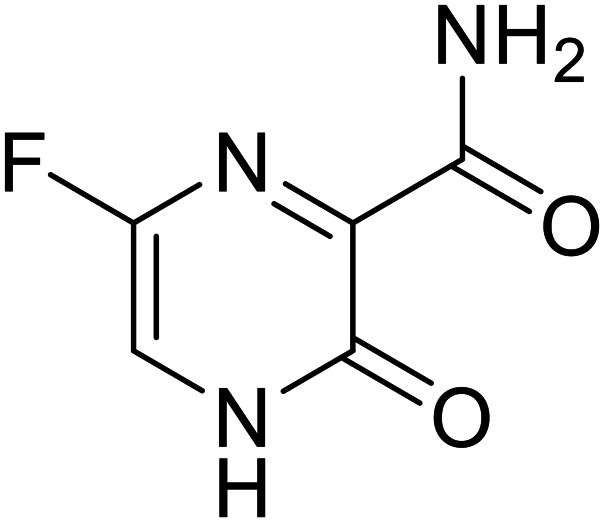	119–123
**Brincidofovir**	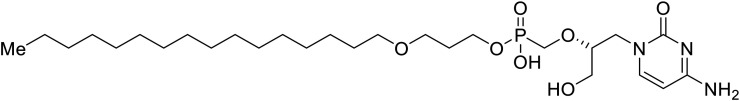	124, 125
9a	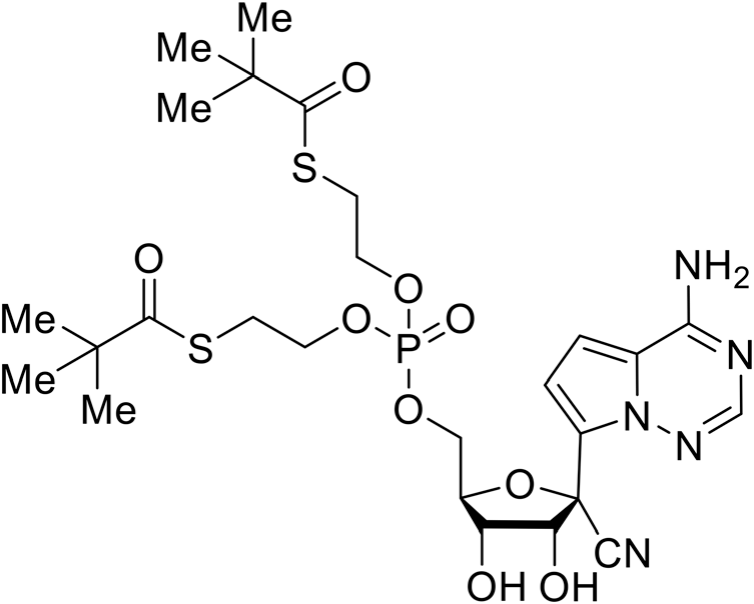	126
**Remdesivir (GS-5734)**	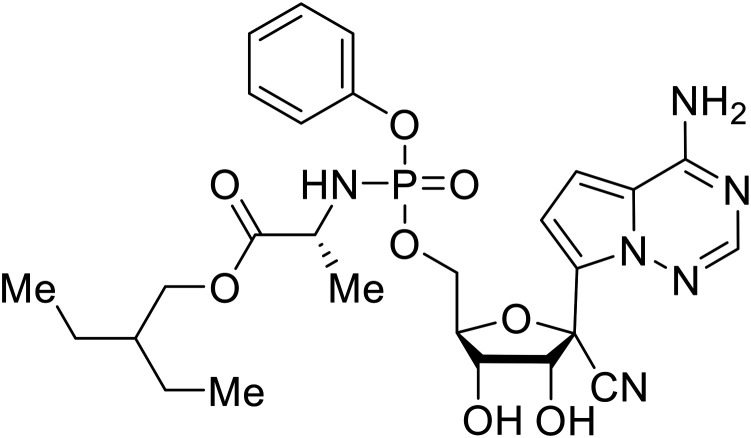	66, 127–130
**RYL-687**	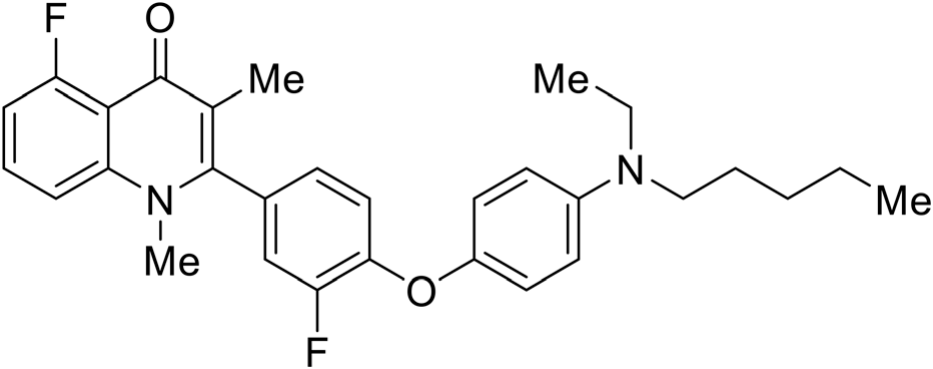	131, 132
**Obeldesivir**	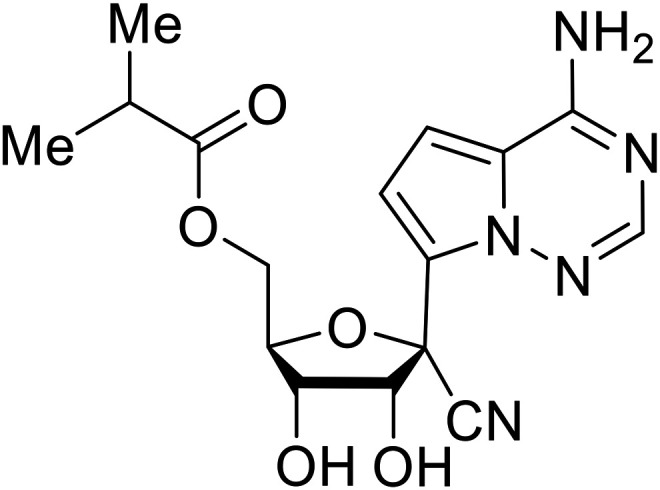	133
**Tolcapone**	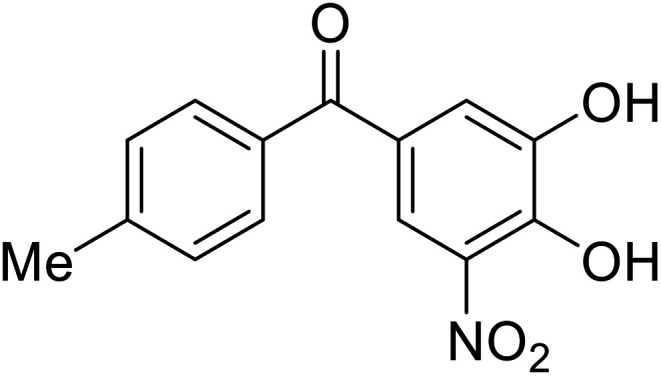	135
**Embelin**	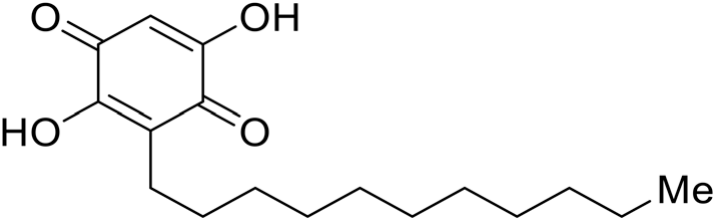	137
**Kobe2602**	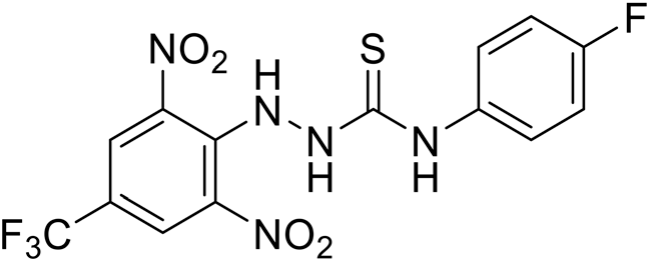	137

**Galidesivir** (**BCX4430**) was designed and synthesized as a novel nucleoside analog to inhibit viral RNA polymerase by premature RNA chain termination during replication and transcription.^117,118^ High content image-based (HCI) assays demonstrated *in vitro* inhibition of recombinant EBOV and SUDV by **galidesivir** (EC_50_ = 11.8 μM and 3.4 μM, respectively). When administered to mice twice daily *via* IM injection or orally, **galidesivir** treatment provided high survival rates among infected mice. **Favipiravir** (**T-705**) is a nucleoside prodrug^119^ previously used as an influenza RNA polymerase inhibitor.^120^ When assessed for EBOV activity, **favipiravir** conferred 100% survival when orally administered to A129 alpha/beta knockdown mice challenged with EBOV.^121^*In vivo* protection against SUDV-challenged guinea pigs was also demonstrated for **favipiravir**.^122^ During the JIKI clinical trial in Guinea, **favipiravir** failed to achieve targeted plasma concentration in patients infected with EBOV, suggesting the need to establish safe dosing ranges in healthy patients prior to efficacy studies.^123^ Similar results occurred for **brincidofovir**, a lipid conjugate of a nucleotide analog prioritized for clinical trials during the 2013–2016 EBOV epidemic.^124,125^ The phase 2 trial consisting of 4 patients with confirmed EBOV infections was inconclusive, as the small sample size confounded **brincidofovir** protection of patients against lethality. The results of **favipiravir** and **brincidofovir** clinical trials exemplified the importance of thorough *in vitro*, *in vivo*, and phase 1 clinical trial data throughout the drug development process.

During SAR development of 1'substituted 4-aza-7,9-dideazaadenosine C-nucleoside analogs, the bis *S*-acyl-2-thioethyl (SATE) prodrug of nucleoside GS-441524 (9a) was synthesized.^126^ Maintaining the prodrug approach to the adenosine analog, **remdesivir** (**GS-5734**) was generated and demonstrated RNA chain terminator activity in its active triphosphorylated form. **Remdesivir**'s selective inhibition of viral RNA polymerase over host polymerase *via* the cyano group^127^ proved advantageous in protecting 100% of rhesus monkeys against infectious EBOV, even when administered 3 days post infection.^128^ Additionally, **remdesivir** protected non-human primates against SUDV infection.^129^ During the EBOV epidemic PALM clinical trial, **remdesivir** was assessed alongside ZMapp, MAb114, and REGN-EB3; however, antibodies MAb114, and REGN-EB3 were more effective compared to **remdesivir**.^66^ Emerged mutations within the EBOV RNA polymerase conferred resistance to **remdesivir**,^130^ resulting in the development of **RYL-687**,^131^ a synthetic analog of RYL-634 that inhibits dihydroorotate dehydrogenase (DHODH) in *de novo* pyrimidine synthesis.^132^ When administered to EBOV ΔVP30 EGFP infected Huh7 cells, **RYL-687** (IC_50_ = 6.65 nM) displayed increased inhibitory EBOV potency compared to **remdesivir** (IC_50_ = 46.6 nM). Continued studies of the GS-441524 nucleoside led to the generation of **obeldesivir**, the isobutyl ester prodrug with improved oral bioavailability,^133^ a favorable attribute to newly developing therapeutics for epi- and pan-demic pathogens. Potent *in vitro* activity of **obeldesivir** against EBOV, SUDV, and MARV led to a non-human primate animal study, where 100% of animals survived lethal SUDV infection upon receiving daily oral **obeldesivir** doses for 10 days. Recent phase 1 trials of healthy patients receiving various doses of **obeldesivir** is advantageous for future **obeldesivir** human efficacy studies.

Most of the replication and transcription inhibitors described above demonstrate an advantageous selectivity for the viral RNA polymerase over host polymerases; however, through their inhibition mechanisms, these small molecules also exhibit activity against other RNA viruses. For filoviral selective replication and transcription inhibitors, an alternative approach to nucleoside analogs or enzyme inhibitors is needed. Studies of the EBOV transcription complex determined a binding event between EBOV's nucleoprotein (NP) and the N-terminus of VP35 at the NPBP binding region. A peptide targeting NPBP was shown to block the NP-VP35 binding event,^134^ which led to the screening of small molecules that could also inhibit this interaction.^135^ Small molecule **tolcapone** was identified as a hit and dose-dependently inhibited EBOV NP-NPBP binding in both fluorescence anisotropy and biolayer interferometry (BLI) experiments. **Tolcapone** also demonstrated inhibitory activity against SUDV, RESTV, and MARV, showcasing pan-filoviral activity; however, additional studies are needed to determine non-filoviral activity. Similarly, the crystal structure of an EBOV NP-derived peptide in complex with VP30 was determined, indicating an alternative approach to potential transcription inhibition.^136^ A fluorescence anisotropy high-throughput screen identified **Embelin** and **Kobe2602** as protein-targeting hits with confirmed VP30 binding as determined by SPR (*K*_D_ = 4.62 μM and 0.88 μM, respectively) and thermal shift assays.^137^ Importantly, a minigenome assay demonstrated transcription inhibition for **Embelin** and **Kobe2602** (EC_50_ = 16.9 μM and 22.3 μM, respectively). Both drugs are expected to target the EBOV NP-VP30 binding, yet **Embelin** and **Kobe2602** are proposed to distinctly bind VP30 at alternate binding regions. Diversified VP30-targeting mechanisms is advantageous for discovering novel binding inhibitors. **Embelin** is an antioxidant while **Kobe2602** is a Ras-binding protein; therefore, both drugs have additional biological targets outside of the EBOV transcriptional complex. For EBOV-selective activity, in-depth SAR exploration is needed to reduce off-target effects; however, both **Embelin** and **Kobe2602**, as well as **tolcapone**, serve as promising starting points to develop novel EBOV protein-targeting replication and transcription inhibitors.

## Budding inhibitors

As packaged viral components are transported to the cell surface, VP40 mediates the budding process. This step is facilitated by VP40's ability to undergo a conformational change from dimeric to hexameric forms. Inhibition of VP40 function by small molecules can block budding required to produce new progeny (Table 7). Computer-based approaches have proven useful to identify potential budding inhibitors. An *in silico* screen of approximately 30 000 Chinese and African natural product-derived small molecules from Northern African Natural Products Database (NANPDB) and traditional Chinese medicine (TCM) were assessed.^138^ A total of 42 naturally derived small molecules with favorable ADMET properties were identified as potential VP40 inhibitors that serve as good starting points for additional experimental analysis. Molecular mechanics and binding free energy calculations were determined for five different reported VP40 inhibitors.^139,140^**Vindesine** possessed the best binding free energy of −5.0 kcal mol^−1^. Within the predicted VP40 binding site, **vindesine** was also hypothesized to from several hydrogen bonding interactions with Gln38, Gln35, and Lys127 that supported its 0.27 μM IC_50_ against VP40. Additionally, **sangivamycin** derived from *Streptomyces* sp. inhibited EBOV VP40 localization to the cell membrane,^141^ which consequently reduced the production and release of eVLPs. As a nucleoside analog, **sangivamycin** also inhibited EBOV transcription and replication in a minigenome assay; therefore, additional studies are required to determine the full mechanism of action of this potential VP40 inhibitor.

**Table 7:** Names, structures, and references of EBOV budding inhibitors

Name	Structure	Ref
**Vindesine**	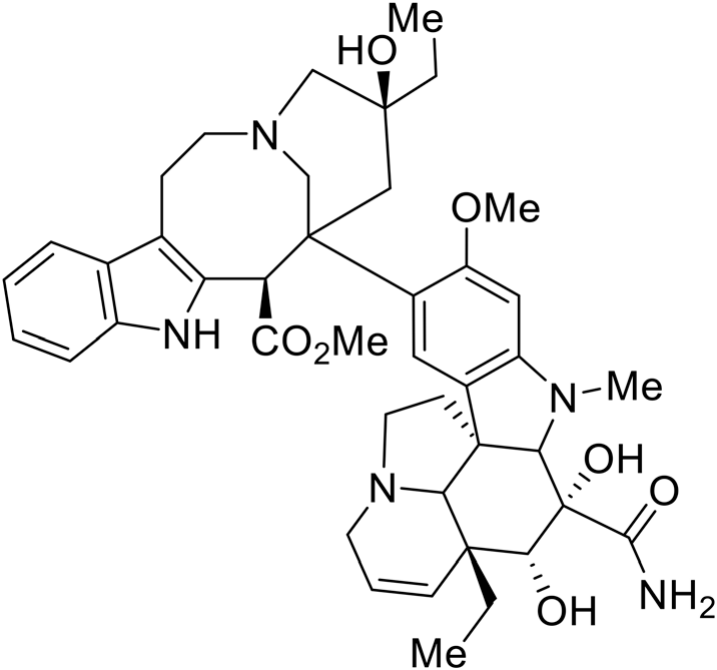	139, 140
**Sangivamycin**	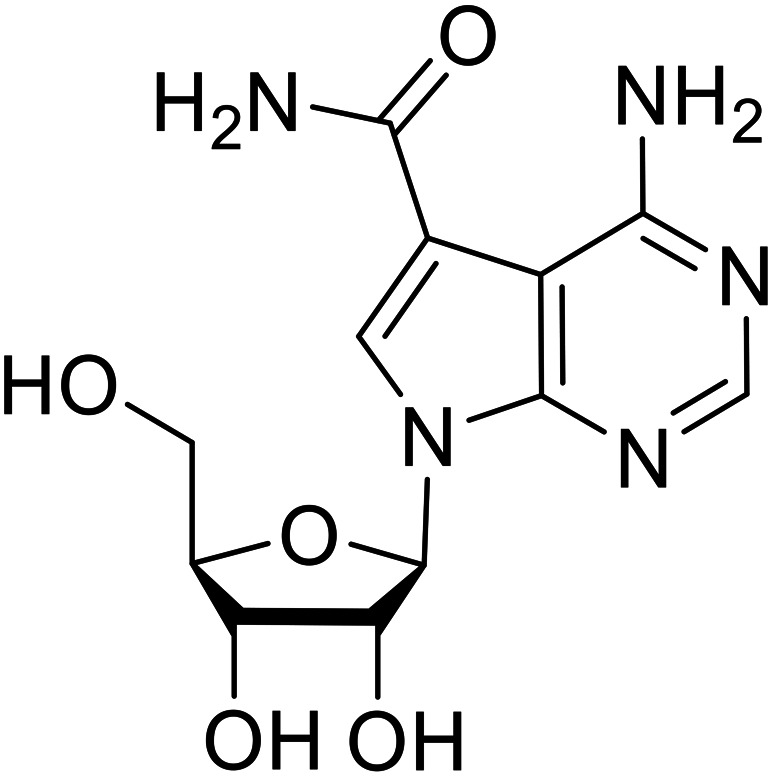	141
**5539-0062**	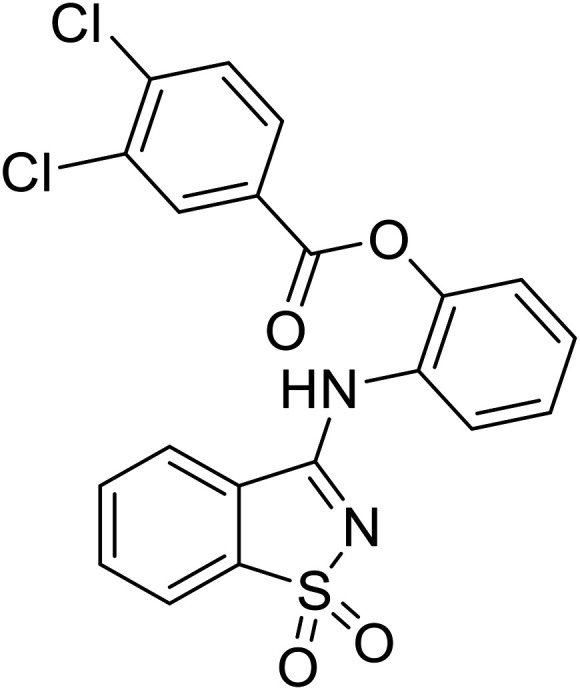	144
4	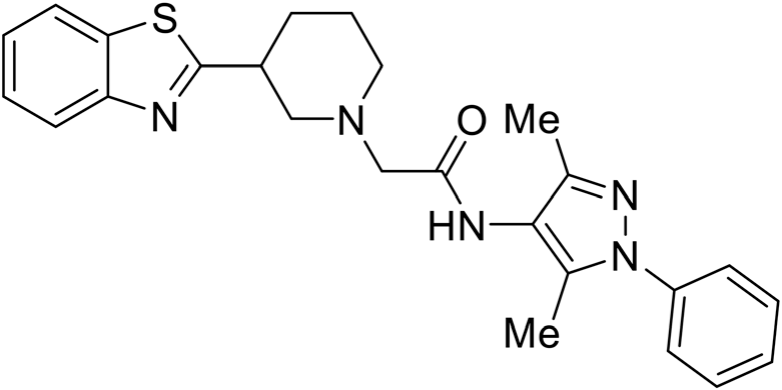	145
5	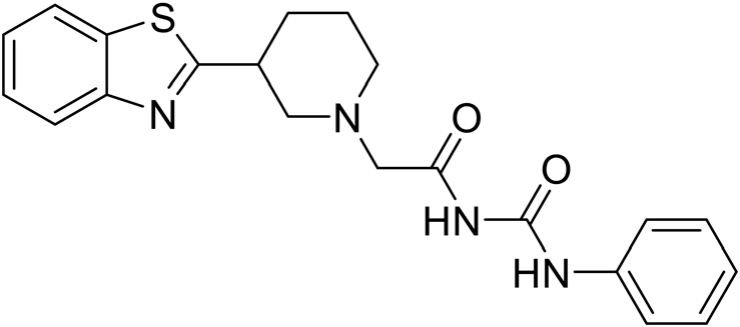	145
21	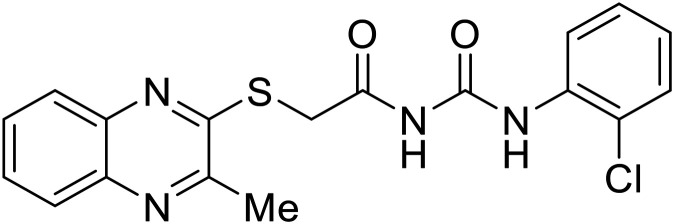	146
24	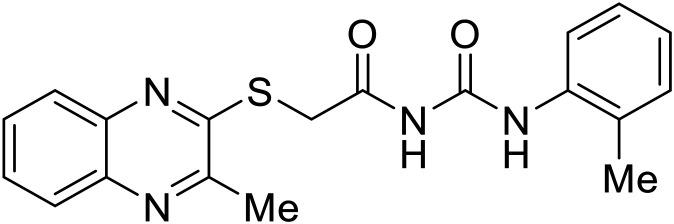	146

EBOV VP40 function is also dependent on interactions made with host proteins within the endosomal sorting complexes required for transport (ESCRT) family. At the proline-rich late (L) domain, VP40 is ubiquitinated by Nedd4 which regulates its interaction with Tsg101.^142,143^ Nedd4 and Tsg101 have thus become potential targets for small molecule budding inhibitors. Biomolecular complementation (BiMC) assays have been used to detect and localize formation of the VP40-Tsg101 and VP40-Nedd4 complexes in cells. Compound **5539-0062** reduced the formation of the EBOV VP40-Tsg101 complex by 76% compared to the DMSO control and further inhibited eVLP egress in HEK 293 T cells.^144^ Similarly, compounds 4 and 5 inhibited the MARV VP40-Nedd4 interaction.^145^ SAR studies of quinoxalin-2-mercapto-acetyl-urea analogs generated potent derivatives 21 and 24 with 2-chlorophenyl and 2-methylphenyl substitutions at the urea site, respectively.^146^ Both 21 and 24 inhibited the MARV VP40-Nedd4 interaction and reduced EBOV VLP formation 93% and 83%, respectively, at 30 nM. Additional human liver microsome stability supports the continued study of compounds 21 and 24 as potential EBOV budding inhibitors.

## Conclusions

The re-emergence of EBOV outbreaks, ease of transmission, EVD prognosis, and lethality continue to stress the need for available EBOV therapeutics. The current FDA-approved monoclonal antibody treatments, although effective, are limited in their use and practicality. Small molecules presented in this literature review have described essential studies that discovered fundamental aspects of the filoviral life cycle, as well as the recent development of effective small-molecule treatments for EBOV.

Although great advances have been made, progress remains limited. Many of the antifiloviral treatments described above have focused on defining the mechanism of action and improving the potency of the small molecules discovered, but have not addressed future steps on transforming the active agents into actual drugs. Furthermore, much of the biological assays assessing activity was conducted *in vitro*, which is far removed from clinically supportive data. For more progressive outcomes in the early drug discovery pipeline, SAR studies should define pharmacokinetics, including metabolic stability, bioavailability, and distribution, and establish strategies for *in vivo* studies. With improved computational tools for structure-based design, SAR-focused studies could also provide more insights to how proposed or experimental chemical modifications improve activity and drug-like properties. Additionally, future work should also monitor drug resistance through serial passaging experiments to identify potential mutations that may arise during outbreaks. Plans to assess newly designed small molecules and combination therapies to address resistance should be proposed for future studies. With continued efforts and strides to advance the current state of small molecule antifilovirals, these active agents have the promise of becoming effective therapeutics.

## Conflicts of interest

The authors declare the following competing financial interest(s): L. R. is the owner of Chicago BioSolutions, Inc. and thus declares potential financial interests, as does I. N. G. who are employed by Chicago BioSolutions, Inc.

## Data Availability

The data in this publication have been previously described in the literature. No new data have been reported in this manuscript.
